# Searching for spin glass ground states through deep reinforcement learning

**DOI:** 10.1038/s41467-023-36363-w

**Published:** 2023-02-09

**Authors:** Changjun Fan, Mutian Shen, Zohar Nussinov, Zhong Liu, Yizhou Sun, Yang-Yu Liu

**Affiliations:** 1 College of Systems Engineering, National University of Defense Technology, 410073 Changsha, China; 2 Department of Physics, Washington University in St. Louis, Campus Box 1105, 1 Brookings Drive, St. Louis, MO 63130 USA; 3 Rudolf Peierls Centre for Theoretical Physics, University of Oxford, Oxford, OX1 3PU UK; 4 LPTMC, Sorbonne Université, Paris, 75006 France; 5 Department of Computer Science, University of California, Los Angeles, CA 90024 USA; 6 Channing Division of Network Medicine, Department of Medicine, Brigham and Women’s Hospital and Harvard Medical School, Boston, MA 02115 USA; 7 Center for Artificial Intelligence and Modeling, The Carl R. Woese Institute for Genomic Biology, University of Illinois at Urbana-Champaign, Champaign, IL 61820 USA

**Keywords:** Computational science, Statistical physics

## Abstract

Spin glasses are disordered magnets with random interactions that are, generally, in conflict with each other. Finding the ground states of spin glasses is not only essential for understanding the nature of disordered magnets and many other physical systems, but also useful to solve a broad array of hard combinatorial optimization problems across multiple disciplines. Despite decades-long efforts, an algorithm with both high accuracy and high efficiency is still lacking. Here we introduce DIRAC – a deep reinforcement learning framework, which can be trained purely on small-scale spin glass instances and then applied to arbitrarily large ones. DIRAC displays better scalability than other methods and can be leveraged to enhance any thermal annealing method. Extensive calculations on 2D, 3D and 4D Edwards-Anderson spin glass instances demonstrate the superior performance of DIRAC over existing methods. The presented framework will help us better understand the nature of the low-temperature spin-glass phase, which is a fundamental challenge in statistical physics. Moreover, the gauge transformation technique adopted in DIRAC builds a deep connection between physics and artificial intelligence. In particular, this opens up a promising avenue for reinforcement learning models to explore in the enormous configuration space, which would be extremely helpful to solve many other hard combinatorial optimization problems.

## Abstract (web summary)

Finding the ground states of spin glasses relevant for disordered magnets and many other physical systems is computationally challenging. The authors propose here a deep reinforcement learning framework for calculating the ground states, which can be trained on small-scale spin glass instances and then applied to arbitrarily large ones.

## Introduction

The Ising spin glass is a classical disordered system that has been studied for decades^1,2^. Its spectacular behaviors have attracted considerable interests in several branches of science, including physics, mathematics, computer science, and biology. The endogenous nature of quenched disorder in spin glasses results in the fact that, it is hard to find out the ground state of such a system due to the frustrations (i.e., the impossibility of simultaneously minimizing all the interactions), despite its seemingly simple Hamiltonian^3^:

1 $${{{{{{{\mathcal{H}}}}}}}}=-\mathop{\sum}\limits_{\langle i,j\rangle }{J}_{ij}{\sigma }_{i}{\sigma }_{j}.$$

**Fig. 1:**
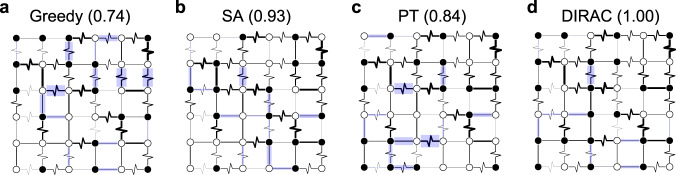
Case study comparison. We applied four algorithms: (**a**) Greedy, (**b**) Simulated annealing (SA), (**c**) Parallel tempering (PT), and (**d**) DIRAC (more precisely, DIRAC^*m*^) to compute the ground state of a randomly generated 6 × 6 Edwards-Anderson (EA) spin glass instance with fixed boundary conditions and couplings *J*_*ij*_ sampled from the Gaussian distribution ${{{{{{{\mathcal{N}}}}}}}}(0,1)$. The ferromagnetic bonds (*J*_*ij*_ > 0) are shown with straight lines, while the anti-ferromagnetic bonds (*J*_*ij*_ < 0) are shown with zigzag lines. The width of the lines are proportional to ∣*J*_*ij*_∣. Nodes are filled/hollow if the spin values *σ*_*i*_ = +1/−1, respectively. If the energy of a bond, − *J*_*ij*_*s*_*i*_*s*_*j*_, is positive, namely not satisfied, we draw a light blue shaded rectangle around the bond, with width proportional to ∣*J*_*ij*_∣. This way a smaller total shaded area of the image corresponds to a lower system energy. We also showed the approximate ratio (prediction/ground truth, where the numerator is the energy of the predicted ground state computed by each method and the denominator is the exact ground state energy computed by Gurobi, a branch-and-bound based exact solver) in brackets. Note that in this small case DIRAC has actually achieved the exact ground state.

In general, this Hamiltonian can be defined on arbitrary graphs. Here, we will focus on the most heavily studied lattice realization of the nearest neighbor Ising spin glass, where the sites lie on a *D*-dimensional hypercubic lattice with *N* = *L*^*D*^ sites (see Fig. 1 for 2D instances) and *σ*_*i*_ = ± 1 represents the binary Ising spin value at site *i*. The coupling *J*_*ij*_ is a Gaussian random variable that represents the interaction strength between two neighboring spins *i* and *j*. In the literature, this is often referred to as the Edwards-Anderson (EA) spin glass model. The EA model aims at capturing the quintessential character of real, physically occurring, spin glasses^4^. Comparing to other short range models such as the mean-field Bethe Lattice^5^, the EA model seems more challenging in the sense that there exists vast amounts of short loops that will lead to much more frustrations.

There are at least three strong motivations to find the ground states of spin glasses. First of all, finding the spin glass ground states is a key to the mysteries behind the strange and complex behaviors of spin glasses (and many other disordered systems), such as its glassy phase^6^ and ergodicity breaking^7^. In particular, ground-state energies in different boundary conditions can be used to compute the stiffness exponent of spin glasses, which can help us ensure the existence of a spin glass phase at finite temperatures^8,9^. Second, finding ground states of Ising spin glasses in three or higher dimensions is a non-deterministic polynomial-time (NP) hard problem^10^, which is closely related to many other hard combinatorial optimization problems^11^. For example, all of Karp’s 21 NP-complete problems and many NP-hard problems (such as the max-cut problem, the traveling salesman problem, the protein folding problem, etc.) have Ising spin glass formulations^11–13^. Therefore, finding the Ising spin glass ground states may help us solve many other NP-hard problems. Finally, the celebrated Hopfield model^14^ and other pioneering models of neural networks drew deep connections with Ising magnets^15^ (and spin glasses, in particular^16,17^) on general networks. The study of spin glasses and their ground states has led to (and will continue lead to) the development of powerful optimization tools such as the cavity method and Belief Propagation that will further shed new light on computational complexity transitions^2,18^.

Given the NP-hard nature of finding the spin glass ground states in three or higher dimensions, the exact branch-and-bound approach can only be used for very small systems^19^. For two-dimensional lattices with periodic boundary conditions in at most one direction (or planar graphs in general), the Ising spin glass ground states can be calculated by mapping to the minimum-weight perfect matching problem, which can be exactly solved in polynomial time^20,21^. However, for general cases with large system sizes, we lack a method with both high accuracy and high efficiency. We used to rely on heuristic methods. In particular, Monte Carlo methods based on thermal annealing, e.g., simulated annealing (SA)^22^, population annealing^23^ and parallel tempering (PT)^24–27^, have been well studied in the statistical physics community.

Recently, reinforcement learning (RL) has proven to be a promising tool in tackling many combinatorial optimization problems, such as the minimum vertex cover problem^28^, the minimum independent set problem^29^, the network dismantling problem^30^, the travelling salesman problem^31^, the vehicle routing problem^32^, etc. Compared to traditional methods, RL-based algorithms are believed to achieve a more favorable trade-off between accuracy and efficiency. We note that RL was recently used to devise a smart temperature control scheme of simulated annealing in finding ground states of the 2D spin glass system, which enabled small systems to better escape local minimum and reach their ground states with high probability^33^. However, this RL-enhanced simulated annealing still fails in finding ground states for larger spin glass systems in three or higher dimensions.

In this work, we introduce DIRAC (Deep reinforcement learning for spIn-glass gRound-stAte Calculation), a RL-based framework that can directly calculate spin glass ground states. DIRAC has several advantages. First, it demonstrates superior performances (in terms of accuracy) over the state-of-the-art thermal annealing methods, especially when the gauge transformation (GT) technique is adopted in DIRAC. Second, it displays better scalability than other methods. Finally, it can be leveraged to enhance any thermal annealing method and offer much better solutions.

## Results

### Reinforcement learning formulation

Following many other RL formulations in solving combinatorial optimization problems^31,34–36^, DIRAC considers the spin glass ground state search as a Markov decision process (MDP), which involves an agent interacting with its environment (i.e., the input instance), and learning an optimal policy that sequentially takes the long-sighted action so as to accumulate its maximum rewards. To better describe this process, we first define state, action and reward in the context of Ising spin glass ground state calculation. State: a state *s* represents the observed spin glass instance, including both the spin configuration {*σ*_*i*_} and the coupling strengths {*J*_*ij*_}, based on which the optimal action will be chosen. The terminal state *s*_T_ is met when the agent has tried to flip each spin once. Action: an action *a*^(*i*)^ means to flip spin *i*. Reward: the reward $r(s,{a}^{(i)},{s}^{{\prime} })$ is defined as the energy change after flipping spin *i* from state *s* to get a new state ${s}^{{\prime} }$, i.e., $r(s,{a}^{(i)},{s}^{{\prime} })=2{\sum }_{j\in \partial i}{J}_{ij}{\sigma }_{i}{\sigma }_{j}$, where ∂*i* represents the set of nearest neighbors of spin *i*.

Through the RL formulation, we seek to learn a policy *π*_Θ_(*a*^(*i*)^∣*s*) that takes any observed state *s* and produces the action *a*^(*i*)^ corresponding to the optimal spin flip that maximizes the expected future cumulative rewards. Here ${{\Theta }}=\{{{{\Theta }}}_{{{{{{{{\mathcal{E}}}}}}}}},{{{\Theta }}}_{{{{{{{{\mathcal{D}}}}}}}}}\}$ represents a collection of learnable encoding parameters ${{{\Theta }}}_{{{{{{{{\mathcal{E}}}}}}}}}$ and decoding parameters ${{{\Theta }}}_{{{{{{{{\mathcal{D}}}}}}}}}$, which will be updated through RL.

### DIRAC architecture

**Fig. 2:**
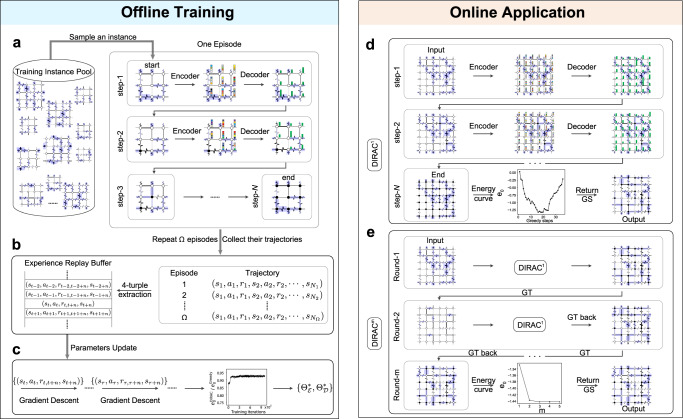
Overview of DIRAC. The DIRAC framework consists of two phases: offline training and online application. (*Left*) During training, we first generate random small EA spin glass instances with couplings sampled from Gaussian distribution, as the training data. **a** For each episode, we sample a random instance of size *N*, and let DIRAC learn to find its ground state. During each episode, the agent starts from the all-spins-up configuration and ends at the all-spins-down configuration, with each spin flipped only once. For the next episode, we sample another training instance. To determine the right action to take, DIRAC first adopts an encoder to represent each node as an embedding vector (shown as a color bar), and then decodes a *Q*-value (shown as a green bar with heights proportional to its value) for each node that predicts its long-term gain. **b** When one episode ends, we collect the trajectory (*s*_1_, *a*_1_, *r*_1_, …, *s*_*N*_) generated during this process, extract the 4-tuple transitions, i.e., (*s*_*t*_, *a*_*t*_, *r*_*t*,*t*+*n*_, *s*_*t*+*n*_), where ${r}_{t,t+n}=\mathop{\sum }\nolimits_{k=t}^{t+n}{\gamma }^{k}{r}_{k}$, and push them into the experience replay buffer ${{{{{{{\mathcal{B}}}}}}}}$, which is a queue that maintains *S*_buffer_ most recent *n*-step transitions. **c**, To update parameters ${{\Theta }}=\{{{{\Theta }}}_{{{{{{{{\mathcal{E}}}}}}}}},{{{\Theta }}}_{{{{{{{{\mathcal{D}}}}}}}}}\}$, we randomly sample mini-batch transitions from the buffer and perform gradient descents over Eq. (3). Repeat this process until the number of training episodes reaches Ω = 10^6^. The best model is selected with achieving the highest validation performance, which is measured by the approximation ratio (${e}_{0}^{{{{{{{{\rm{DIRAC}}}}}}}}}/{e}_{0}^{{{{{{{{\rm{Greedy}}}}}}}}}$) on the validation data. Here ${e}_{0}^{{{{{{{{\rm{DIRAC}}}}}}}}}$ and ${e}_{0}^{{{{{{{{\rm{Greedy}}}}}}}}}$ are the energy densities computed by DIRAC and by Greedy respectively. (*Right*) During application, we have two basic DIRAC strategies: DIRAC^1^ and DIRAC^*m*^. **d**, For an input instance, DIRAC^1^ (with the optimized parameters $\{{{{\Theta }}}_{{{{{{{{\mathcal{E}}}}}}}}}^{*},{{{\Theta }}}_{{{{{{{{\mathcal{D}}}}}}}}}^{*}\}$) starts from {*σ*_*i*_ = + 1}, and greedily flips the highest-*Q* spins till {*σ*_*i*_ = − 1}. The spin configuration of the lowest energy encountered during this process is returned as the predicted ground state. **e**, DIRAC^*m*^ refers to a sequential running of *m* iterations of DIRAC^1^ connected by GTs. For each iteration, GT converts the lowest-energy configuration from last iteration to be {*σ*_*i*_ = + 1} for DIRAC^1^, and convert it back as the output of the current iteration.

We design DIRAC to learn the policy *π*_Θ_ automatically. As shown in Fig. 2, DIRAC consists of two phases: offline training and online application. For offline training, the DIRAC agent is self-taught on randomly generated small-scale EA spin glass instances. For each instance, the agent interacts with its environment through a sequence of states, actions and rewards (Fig. 2a). Meanwhile, the agent gains experiences to update its parameters, which enhances its ability in finding the ground states of EA spin glasses (Fig. 2b,c). For online application, the well-trained DIRAC agent can be used either directly (DIRAC^1^, Fig. 2d) or iteratively (DIRAC^*m*^, Fig. 2e) or just as a plug-in to a thermal annealing method (DIRAC-SA and DIRAC-PT), on EA spin glass instances with much larger sizes than the training ones.

DIRAC’s success is mainly determined by the following two key issues: (1) How to represent states and actions effectively? (2) How to leverage these representations to compute a *Q*-value, which predicts the long-term gain for an action under a state. We refer to these two questions as the encoding and decoding problem, respectively.

#### Encoding

**Fig. 3:**
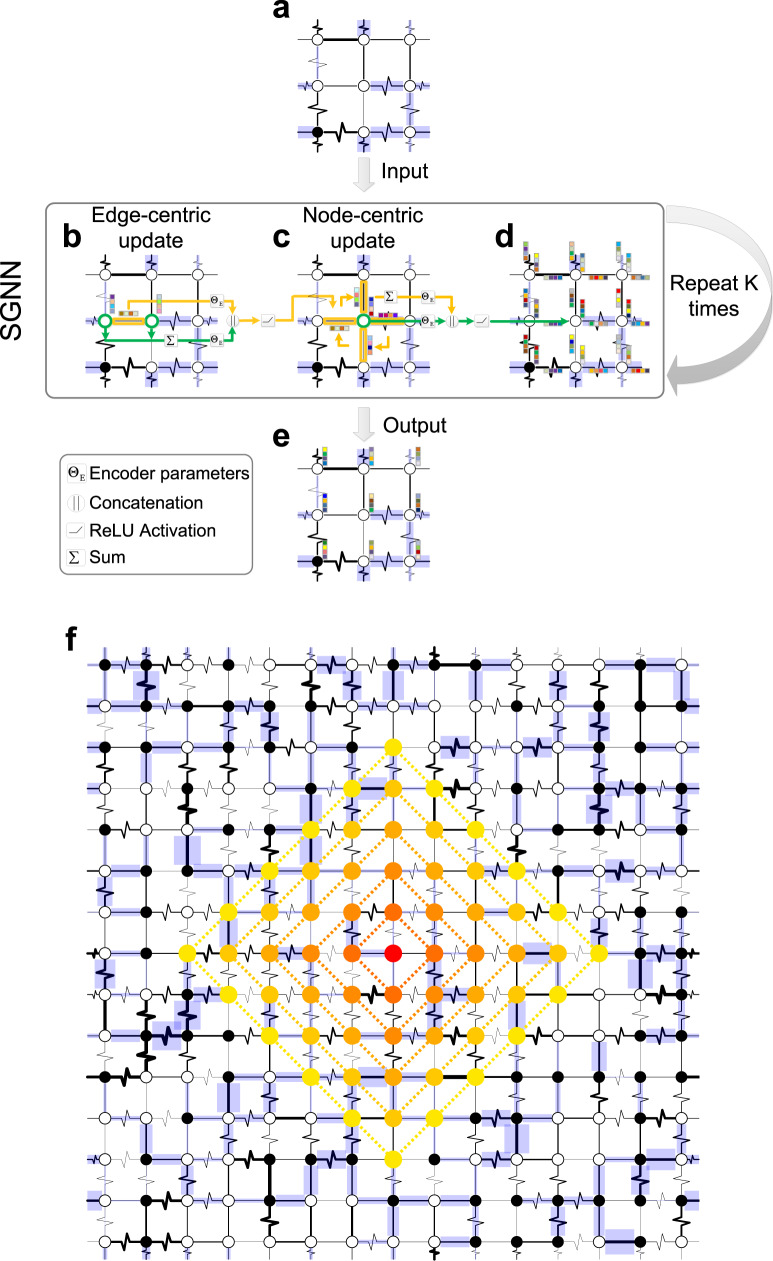
The encoder Spin Glass Neural Network (SGNN). SGNN encodes (**a**) the input spin glass instance (with any arbitrary spin configuration) into a (**e**) low-dimensional space, where each spin is associated with an embedding vector (shown as a color bar). **b** SGNN first updates edge embedding vectors based on the edge itself and its adjacent nodes, and then (**c**) updates node embedding vectors based on the node itself and its adjacent edges. Note that the node-centric updates take place only when the edge-centric updates finish for all edges at each layer. Both updates are followed by a non-linear transformation operator (e.g., ReLU) with learnable parameters. The edge features are initialized by edge weights, and the node features are initialized by its coordinates in the hypercubic coordinate system. **d**, Each node or edge updates its embedding vector in one layer. Repeating several layers, we obtain an embedding vector for each node (**e**) that reflects its informative features. **f**, Each layer of updates increases the long-range couplings with one more hop’s neighbors (dashed lines) for a given spin. For example, for the central spin (colored in dark red), its final embedding vector after *K* = 5 layers captures both its position and its long-range couplings with neighbors within *K* = 5 hops.

Since a hypercubic lattice can be regarded as a special graph, we design an encoder based on graph neural networks^37–41^, namely SGNN (Spin Glass Neural Network), to represent states and actions. As shown in Fig. 3, to capture the coupling strengths {*J*_*ij*_}, which are crucial to determine the spin glass ground states, SGNN performs two updates at each of the *K* iterations: the edge-centric update and the node-centric update, respectively. Here,the hyper-parameter *K* represents the number of message-passing steps in SGNN, and we set *K* = 5 in our calculations. The edge-centric update (Fig. 3b, Fig. S1a) aggregates edge embedding vectors, which are initialized as edge input features (SI Sec. IA), from its adjacent nodes. The node-centric update (Fig. 3c, Fig. S1b) aggregates node embedding vectors, which are initialized as node input features (SI Sec. IA), from its adjacent edges. Both updates concatenate the self embedding and the neighborhood embedding and are then subjected to a non-linear transformation (e.g., rectified linear unit, ${{{{{{{\rm{ReLU}}}}}}}}(z)=\max (0,z)$). Traditional graph neural networks architectures often carry only node-centric updates^37–39,41^, with edge weights taken as node’s neighborhood if needed. Yet this would fail in our case where edge weights play vital roles, and lead to unsatisfactory performances (see ablation study in SI Sec. IF and Fig. S2).

SGNN repeats *K* iterations of both edge-centric and node-centric updates, and finally obtains an embedding vector for each node (or spin) (Fig. 3e). Essentially, each node’s embedding vector after *K* iterations captures both its position in the lattice and its long-range couplings with neighbors within *K* hops (see Fig. 3f for an example of *K* = 5). In our RL setting, each node is subject to a potential action, thus we also call the embedding vector of node *i*, denote as *z*_*i*_, its action embedding. Collectively, we denote *z*_*a*_ = {*z*_*i*_}, which includes embedding vectors for all the nodes *i* = 1, ⋯ , *N*. To represent the whole lattice (i.e., the state in our setting) and obtain the state embedding, denote as *z*_*s*_, we sum over all node embedding vectors^41^, which is a straightforward and empirically effective way for graph-level encoding. (SI Sec. IA and Algo. S3 describe more details about SGNN.)

#### Decoding

Once the action embeddings *z*_*a*_ and state embedding *z*_*s*_ have been computed, DIRAC will leverage these representations to compute the state-action pair value function *Q*(*s*, *a*^(*i*)^; Θ), which predicts the expected future cumulative rewards if taking action *a*^(*i*)^ under state *s*, and following the policy *π*_Θ_(*a*^(*i*)^∣*s*) till the end of the episode (i.e., till all the spins have been flipped once). Hereafter, we will refer to this function as the *Q*-value of spin-*i*. Specifically, we concatenate the embeddings of state and action, and apply a neural network with non-linear transformations to map the concatenation [*z*_*s*_, *z*_*i*_] to a scalar value. In theory, any neural network architecture can be used. Here for the sake of simplicity, we adopt the classical multilayer perceptron (MLP) with ReLU activation. (see SI Sec. IB for more details):

2 $$Q(s,{a}^{(i)};{{\Theta }})={{{{{{{\rm{MLP}}}}}}}}([{{{{{{{{\bf{z}}}}}}}}}_{s},{{{{{{{{\bf{z}}}}}}}}}_{i}];{{\Theta }}).$$

Note that here ${{\Theta }}=\{{{{\Theta }}}_{{{{{{{{\mathcal{E}}}}}}}}},{{{\Theta }}}_{{{{{{{{\mathcal{D}}}}}}}}}\}$, ${{{\Theta }}}_{{{{{{{{\mathcal{E}}}}}}}}}$ are the SGNN encoder parameters (see SI Eq. 1–Eq. 2), ${{{\Theta }}}_{{{{{{{{\mathcal{D}}}}}}}}}$ are the MLP decoder parameters (see SI Eq. 3).

#### Offline training

We will adopt the above *Q* function to calculate the spin glass ground state. Prior to that, we first need to optimize the *Q* function to predict a more accurate future gain.

We define the *n*-step *Q*-learning loss as:

3 $${{{{{{{\mathcal{L}}}}}}}}={{\mathbb{E}}}_{({s}_{t},{a}_{t},{r}_{t,t+n},{s}_{t+n}) \sim {{{{{{{\mathcal{B}}}}}}}}}\left[{\left({r}_{t,t+n}+\gamma \mathop{\max }\limits_{{a}_{t+n}}Q({s}_{t+n},{a}_{t+n};\hat{{{\Theta }}})-Q({s}_{t},{a}_{t};{{\Theta }})\right)}^{2}\right],$$

and we perform mini-batch gradient descent to update parameters Θ over large amounts of experience transitions, which are represented by the 4-tuple transitions (*s*_*t*_, *a*_*t*_, *r*_*t*,*t*+*n*_, *s*_*t*+*n*_) in the DIRAC framework. The transitions are randomly sampled from the experience replay buffer ${{{{{{{\mathcal{B}}}}}}}}$, *s*_*t*_ and *a*_*t*_ denote the state and action at time step *t*, respectively. ${r}_{t,t+n}=\mathop{\sum }\nolimits_{k=0}^{n-1}{\gamma }^{k}r({s}_{t+k},{a}_{t+k},{s}_{t+k+1})$ represents the *n*-step accumulated reward, the discount factor *γ* is a hyper-parameter that controls how much to discount future rewards. $\hat{{{\Theta }}}$ is the target parameter set, which will only be updated with Θ every a certain number of episodes (see SI Sec. IC and Algo. S4 for more details on training).

#### Online application

DIRAC is trained over a large number of small random spin glass instances. Once the training phase is finished, we will perform the optimized *Q*-based ground state search. Traditional *Q*-based strategy greedily takes the highest-*Q* action each step till the end. Here we adopt the batch nodes selection strategy^30^, i.e., at each step we flip a top fraction (e.g., 1%) of the spins with highest *Q*-values. Similar to the training phase, we start from the all-spins-up configuration, end at the all-spins-down configuration, and each spin is flipped only once. Hereafter we refer to this process as DIRAC^1^. The spin configuration of the lowest energy encountered during this process is returned as the predicted ground state. Note that starting from the same configuration forces the agent to learn a strategy with the same starting point, which drastically reduces the potential trajectory space and thus requires less data for training. Ending at the same configuration makes the agent always finish the MDP within finite steps. This finite-horizon MDP forces the agent to pick the right move without allowing too many regrets.

We emphasize that the vanilla strategy DIRAC^1^ has several limitations. First, it can only handle one single uniform initialization {*↑*, ⋯ , *↑*}, rather than multiple random initializations. This drastically hinders DIRAC’s performance as significant performance improvements would be achieved by simply taking the best solution found across multiple initializations. Second, starting from the all-spins-up configuration and ending at the all-spins-down configuration (with each spin flipped only once) is certainly not ideal. An ideal way is to let the agent “revisit” its earlier decisions so as to search for an ever-improving solution. After all, due to the inherent complexity of combinatorial optimization problems, a policy that produces only one single “best-guess” solution is often sub-optimal. However, most of the existing RL algorithms are unable to revisit their earlier decisions in solving combinatorial optimization problems, because they often use a greedy strategy to construct the solution incrementally, adding one element at a time. To solve this issue, many recent attempts designed complex neural network architectures with lots of tedious and ad hoc input features^42,43^. Yet, their presented results are far from satisfactory, they could not achieve the exact ground truth on even small instances, and they lack the validations on large instances.

To resolve the limitations of DIRAC^1^, we employ the technique of GT in Ising systems^44^. The GT between one spin glass instance {*σ*_*i*_, *J*_*ij*_} and another instance $\{{\sigma }_{i}^{{\prime} },{J}_{ij}^{{\prime} }\}$ are given by^45,46^:

4 $${J}_{ij}^{{\prime} }={J}_{ij}{t}_{i}{t}_{j},{\sigma }_{i}^{{\prime} }={\sigma }_{i}{t}_{i},$$

where *t*_*i*_ = ± 1 are independent auxiliary variables so that ${\sigma }_{i}^{{\prime} }$ can take a desired Ising spin value. This technique is able to switch the spin glass system between any two configurations while keeping the system energy invariant (since ${J}_{ij}^{{\prime} }{\sigma }_{i}^{{\prime} }{\sigma }_{j}^{{\prime} }={J}_{ij}{\sigma }_{i}{\sigma }_{j}$), which also means any input configuration can be gauge transformed to the all-spins-up configuration. In this way, DIRAC^1^ is able to handle any random input spin configuration. Note that if there exists external fields *h*_*i*_, we only need to make ${h}_{i}^{{\prime} }={h}_{i}{t}_{i}$ so that GT still works.

With the aid of GT, we can design a more powerful strategy beyond DIRAC^1^, referred to as DIRAC^*m*^ hereafter. As the name suggests (also shown in Fig. 2e), DIRAC^*m*^ repeats *m* iterations of DIRAC^1^. During each iteration, DIRAC^1^ starts from an instance with all-spins-up configuration, which is obtained by gauge transforming the lowest-energy configuration found in the previous iteration, until the system energy no longer decreases. (As shown in SI Fig. S3, initializing from the lowest-energy configuration found in the previous iteration is much better than from a random one.) Notably, GT allows DIRAC^*m*^ to revisit the earlier decisions by computing a new set of *Q*-values (so as to re-evaluate the states and actions) at each iteration. The *Q*-value can be seen as a function of {*J*_*ij*_, *σ*_*i*_}, i.e., *Q*(*J*_*ij*_, *σ*_*i*_). DIRAC will generate different *Q*-values for different instances, as long as they have different bond signs and spin values (even if those instances are connected by GTs and hence share the same physics). This also explains why GT only works for DIRAC, but fails for any other energy-based methods, such as Greedy, SA or PT. Those methods only consider the energy of each bond, while GT does not change the energy of each bond at all: $(-{J}_{ij}{\sigma }_{i}{\sigma }_{j}=-{J}_{ij}^{{\prime} }{\sigma }_{i}^{{\prime} }{\sigma }_{j}^{{\prime} })$. (see SI Sec. ID for more details on DIRAC^*m*^).

Another prime use of GT is the so-called gauge randomization^47^, where one may execute many runs (or randomizations) of DIRAC (either DIRAC^1^ or DIRAC^*m*^) for an input spin glass instance, with each run the instance is randomly initialized with a different spin configuration. The configuration of the lowest energy among these runs is then returned as the predicted ground state of the input instance.

DIRAC can also serve as a plug-in to Monte-Carlo based methods, such as SA and PT. The key ingredient of these methods is the so-called Metropolis-Hastings criterion:

5 $$P({{\Delta }}E;\beta )=\min (1,{e}^{-\beta {{\Delta }}E}),$$

which means that the probability of accepting a move with energy change Δ*E* at *β* (indicates the inverse temperature, 1/*T*) is the minimum of 1 and *e*^−*β*Δ*E*^. The move is usually referred to as a small perturbation of the system, and in our case it just means a single-spin flip. At high temperatures, the Metropolis-Hastings criterion tends to accept all possible moves (including “bad” moves with Δ*E* > 0). However, at low temperatures it is more likely to accept those “good” moves that could lower the energy (i.e., with Δ*E* < 0), rendering the move-and-accept iteration more like a greedy search. We refer the process of using the Metropolis-Hastings criterion to accept moves as the energy-based Metropolis-Hastings (EMH) procedure hereafter. The art of these Monte-Carlo based methods, in some sense, is the balance between exploration at high temperatures and exploitation (energy descents) at low temperatures.

The general idea of using DIRAC as a plug-in to Monte-Carlo based methods is to replace the EMH procedure with DIRAC. (Later in this paper we will demonstrate the long-sighted greediness of DIRAC with respective to a purely energy-based greediness.) Specifically, we design a DIRAC-based Metropolis-Hastings (DMH) procedure. At each iteration, we let the systems or replicas choose randomly between DMH and EMH for a more effective configuration search. The DMH procedure uses one DIRAC^1^ (with the assistance of GT) to lower the system energy. When the system energy reaches a local minimum (i.e., Δ*E* = 0), DMH will perturb the spin configuration by flipping each spin with a temperature-dependent probability (SI Eq. 6). When applying this plug-in idea to SA and PT, we obtain DIRAC-SA and DIRAC-PT, respectively. See SI Sec. IH, Algo. S6, Algo. S7 and Fig. S4 for more details about these two DIRAC-enhanced algorithms.

### Performance of finding the ground state

**Fig. 4:**
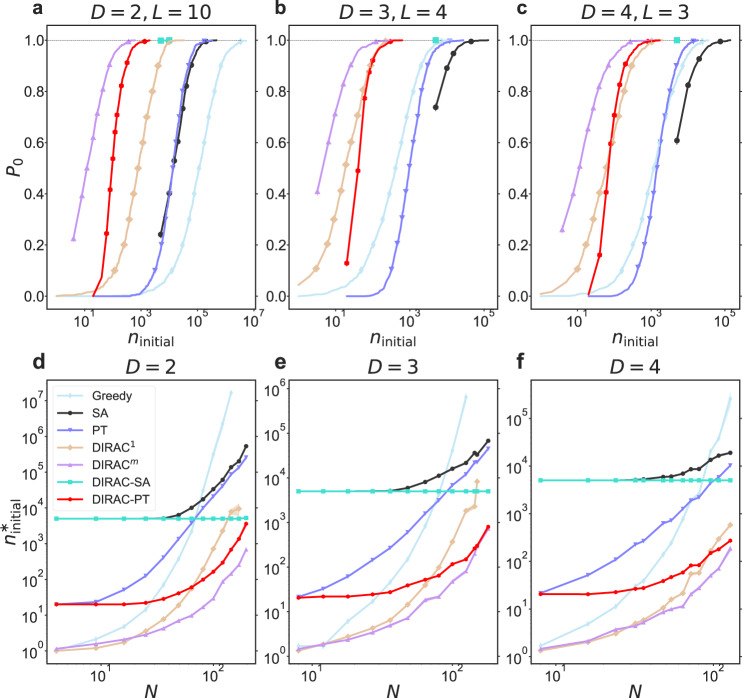
Performance of different methods in finding the ground state. To compare the ability of finding the exact ground states, we evaluated different methods on small spin glass systems for which the true ground state can be computed by the branch-and-bound-based solver Gurobi^49^. In (**a–c**), the quantity we chose to compare is the probability of finding the ground state, denoted as *P*_0_, which is empirically calculated as the fraction of 1000 random instances for which the ground state is found (and confirmed by Gurobi). We computed *P*_0_ as a function of the number of different initial configurations, denoted as *n*_initial_. In (**d–f**), we plot ${n}_{{{{{{{{\rm{initial}}}}}}}}}^{*}$, i.e., the minimum value of *n*_initial_ where *P*_0_ reaches 1, as a function of system size *N*. To compute ${n}_{{{{{{{{\rm{initial}}}}}}}}}^{*}$, we generated 100 random instances for each size, and performed 10 independent runs for each instance. The minimum number of initial configurations that is required by each method to obtain the true ground state was returned as the result of one run. We averaged ${n}_{{{{{{{{\rm{initial}}}}}}}}}^{*}$ over these 1000 independent runs, and showed the results of mean and standard error of the mean (SEM) (shaded area, comparable with the line width or data point symbol size in most cases) of different methods on different sizes. Log scale was used for visualization purpose. Note that to have a variety of system sizes, instead of using the same side *L* for each dimension, we considered different sides for different dimensions. By tuning the sides (*L*_1_, ⋯ , *L*_*D*_), we generated a variety of system size *N* = *L*_1_ × ⋯ × *L*_*D*_. In our implementations, one run of SA and DIRAC-SA needs 5000 initial configurations, and for some of these small systems, one run of DIRAC-SA is able to reach their exact ground states. As a result, the curves of DIRAC-SA are shown to be independent of these presented sizes. Also, the data points for larger *N* fluctuate more because we calculated fewer samples due to the computational resource limits.

To demonstrate the power of DIRAC in finding the ground states of Ising spin glasses, we first calculated the probability *P*_0_ of finding the ground states of small-scale EA spin glass instances as a function of the number of initial spin configurations (denoted as *n*_initial_) (see Fig. 4(a–c)). Here we want to point out the difference between the concepts of initial configuration and run. Usually initial configuration can be seen as the same as sweep, referring to *N* spin-flip (attempts). A run refers to a complete process of an algorithm, and it contains a certain number of initial configurations. For example, PT consists of *N*_e_ epochs and *N*_r_ replicas, in each epoch, a replica will do a single sweep, namely *N* spin-flip attempts, so a run of PT contains *N*_e_ × *N*_r_ initial configurations; DIRAC^*m*^ contains *m* iterations of DIRAC^1^, and each DIRAC^1^ contains *N* spin-flips, so each run of DIRAC^*m*^ is counted as *m* initial configurations. Those instances were chosen to be small so that their exact ground states can be calculated by the branch-and-bound based solver Gurobi within tolerable computing time. For each given *n*_initial_, we empirically computed *P*_0_ as the fraction of 1000 random instances for which the ground state is found by DIRAC (and confirmed by Gurobi^48^). We found that DIRAC enables a much faster finding of ground states than the Greedy, SA, and PT algorithm. In fact, all DIRAC variants (DIRAC^1^, DIRAC^*m*^, DIRAC-SA and DIRAC-PT) facilitate the finding of ground states. For example, in the case of *D* = 2 and *L* = 10 (Fig. 4a), ${n}_{{{{{{{{\rm{initial}}}}}}}}}^{*}$ (the minimum value of *n*_initial_ where *P*_0_ reaches 1.0) of PT is 322,800, while ${n}_{{{{{{{{\rm{initial}}}}}}}}}^{*}$ of DIRAC^*m*^ is only 600. In fact, for DIRAC^*m*^ the ground states can be found with only one gauge randomization for some instances.

To systematically compare those algorithms in terms of their ability of finding the ground states, we investigated the system size scaling of ${n}_{{{{{{{{\rm{initial}}}}}}}}}^{*}$. As shown in Fig. 4(d–f), DIRAC’s superior performances of facilitating the finding of ground states is persistent across different systems with varying sizes, rather than only for the three sizes presented in Fig. 4(a–c).

We notice that the *P*_0_ ~ *n*_initial_ curve of DIRAC-SA contains very few scatter points, and its ${n}_{{{{{{{{\rm{initial}}}}}}}}}^{*}$ is almost independent of the system size *N*. This is because in our implementation, each result of SA or DIRAC-SA is calculated using 5000 initial configurations (one run), and for these small systems, one run of DIRAC-SA is able to reach their ground states. Due to the NP-hard nature of this problem, we believe the ${n}_{{{{{{{{\rm{initial}}}}}}}}}^{*}$ of DIRAC-SA will eventually grow exponentially with *N* for large *N*.

We also notice that in Fig. 4(b,c) the performances of SA and PT seem to be worse than the simple Greedy algorithm. We suspect this is because for those small systems, the simple Greedy algorithm, which greedily flips the highest-energy-drop spin, could reach the ground states much faster than SA or PT. Indeed, those annealing-based algorithms often require multiple energetically-unfavorable spin-flips in order to ultimately reach a lower energy state. For large systems, the Greedy algorithm would easily get stuck in the local minimum and thus need more initial configurations to reach the ground state, as shown in Fig. 4 (a,d–f).

### Performance of minimizing the energy

**Fig. 5:**
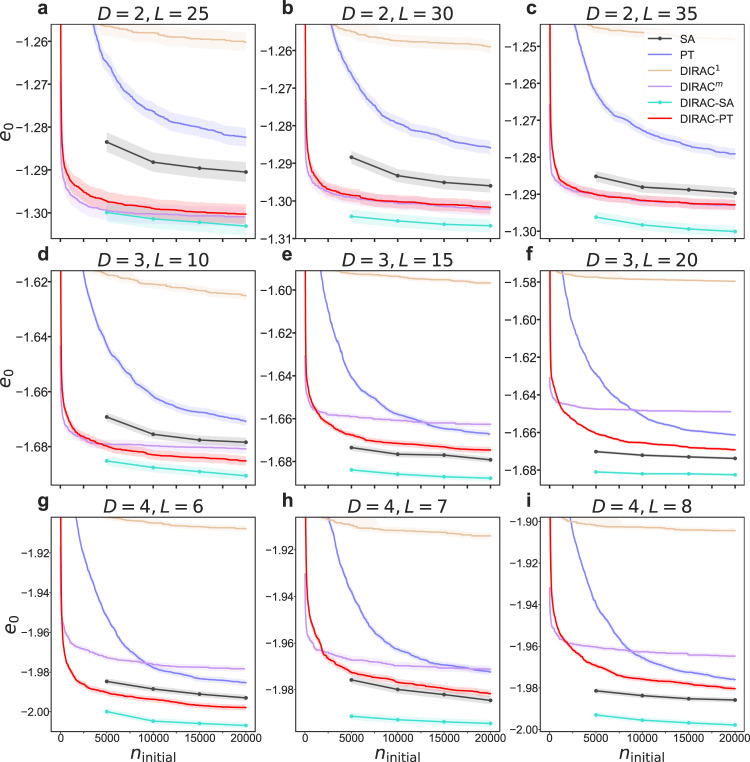
Performance of different methods in minimizing the energy. We compared the disorder averaged “ground-state” energy per spin (predicted by each method), denote as *e*_0_, as a function of the number of initial configurations *n*_initial_, to benchmark various methods on large systems. Since the Greedy algorithm performs the worst in minimizing the energy, its results are not shown here so that we can better compare the performance of other methods. We compared on three dimensions (*D* = 2, 3, 4), and for each dimension we consider three different sides (**a–i**). For each case, we run *n*_initial_ = 2 × 10^4^ initial configurations for each method. At a given *n*_initial_, we chose the lowest energy among all runs for each instance, and averaged the results from 50 independent instances as the final result. We showed their mean and SEM (shaded area) of different algorithms on different sizes. Since each result of SA and DIRAC-SA is calculated using 5000 initial configurations, the curves of SA and DIRAC-SA actually consist of only four scatter points. Note that here the presented ground state energies reached by our PT implementation are different from the one found in the literature. For example, Ref. ^51^ reported an average energy density *e*_0_ = − 1.6981 (PT) for the 3D-10 (*D* = 3, *L* = 10) system, while our PT only reached an average *e*_0_ = − 1.6707 (**f**)). This is simply because different number of initial configurations (*n*_initial_) were used. In Ref. ^51^, the authors used *n*_initial_ = 3.2 × 10^7^ initial configurations to reach *e*_0_ = − 1.6981, while we only used *n*_initial_ = 2 × 10^4^ initial configurations (i.e., fewer than one thousandth of their *n*_initial_) to reach *e*_0_ = − 1.6707. We know that for those annealing methods, the more initial configurations we explored, the more potential to reach a lower energy.

For larger systems, it is hard to compute the probability of finding the ground states for any algorithm, because we need to confirm if the calculated “ground state” is the true ground state obtained by an exact solver, and even the best branch-and-bound solver could not calculate the ground states of very large instances within acceptable time. In this case, a more practical choice of benchmarking various algorithms is to compare the energy of their predicted “ground state”, denoted as *E*_0_, which is not necessarily the true ground state energy, but the lowest energy provided by each algorithm for each particular instance. In particular, we are interested in the disorder averaged “ground-state” energy per spin, denoted as *e*_0_, which is computed as *E*_0_/*N* averaged over many instances. In Fig. 5, we demonstrate *e*_0_ as a function of *n*_initial_ on several large systems. Up to our knowledge, some of these systems, such as *D* = 3, *L* = 20, have never been considered in previous studies. Moreover, we have never seen results on the 4D systems in the literature.

From Fig. 5 we made several following observations. First, DIRAC-SA reaches the lowest *e*_0_ for all cases. In some cases, DIRAC-SA is very close to the reported ground state in existing studies. For example, for *D* = 3 and *L* = 10, Ref. ^49^ reported *e*_0_ = −1.6981 (with *n*_initial_ = 3.2 × 10^7^, obtained by PT), while DIRAC-SA obtained *e*_0_ = − 1.6906 (with *n*_initial_ = 2 × 10^4^, fewer than one thousandth of the number of initial configurations used in Ref. ^49^) (Fig. 5d). Second, the performance of SA in minimizing the system energy is surprisingly good, which is comparable, sometimes even has a better performance than PT (up to *n*_initial_ = 2 × 10^4^). PT has long been considered as the state-of-the-art algorithm for the spin glass ground state problem^27,49,50^. However, our observation suggests that we should revisit the potential of SA in solving this problem. Third, DIRAC as a general plug-in could greatly improve annealing-based Monte-Carlo methods, such as SA and PT. For the nine systems studied in Fig. 5, DIRAC-SA computes an average 0.79% energy lower than SA, and DIRAC-PT calculates an average 2.01% energy lower than PT. Statistical tests indicate that these improvements are not marginal, but statistically significant : *p* value < 10^−4^ for most cases, and < 0.05 for all the cases (Wilcoxon signed-ranked test, see SI, Fig. S5). Finally, there is a clear performance gap between DIRAC^1^ and DIRAC^*m*^ in Fig. 5. This is simply because DIRAC^*m*^ (as a sequential running of *m* iterations of DIRAC^1^ connected by GTs) can jump out of local minimum and finally reaches a much lower energy state than DIRAC^1^.

### Efficiency

Besides the effectiveness, DIRAC is also computationally efficient. For example, during the application phase, at each step DIRAC^1^ flips a small fraction (e.g., 1%) of the highest-*Q* spins, rather than just flipping the spin with the highest *Q*-value as we did in the training phase. In our numerical experiments, we found this batch nodes selection strategy^30^ reduces the running time significantly without sacrificing much accuracy (Fig. S6). Both time complexity analysis (SI Sec. IE, Tab. S1, Fig. S7) and the performance analysis of finding the ground state (Fig. 4) suggest that DIRAC displays a better scalability than other methods.

We should admit that DIRAC needs to be offline trained while other methods needn’t. Yet, we think it is reasonable to compare DIRAC’s efficiency without considering its training time, as DIRAC needs to be trained offline only once for each dimension (Fig. S8), and could then be applied infinite times for the systems (of the same dimension) with different sides. Besides, DIRAC’s training time is often affordable. For some large systems the total costs required by its training and application are still lower than that of PT. For instance, although DIRAC needs about 2.5 h to finish training on the 3D system, it takes only on average 417 s for DIRAC^*m*^ to calculate a random spin glass instance with *D* = 3, *L* = 20 (*n*_initial_ = 10). However, to obtain the same energy, PT needs on average 3 h (*n*_initial_ = 5, 360), which is higher than the total time costs of DIRAC^*m*^ (about 2.62 h) (Fig. 5f). Note that all the calculations were conducted on a 20-core computer server with 512GB memory and a 16GB Tesla V100 GPU.

Since the biggest computational cost of DIRAC is from the matrix multiplications in SGNN, the graphics processing unit (GPU)-accelerations can be more easily applied on DIRAC than other methods, as the matrix multiplication itself is particularly suitable for paralleling. Still, for the sake of fairness, here we report DIRAC’s CPU testing time only, and do not deploy its GPU-accelerations in the application phase. We only utilized GPU to speed up the training process. Hence, the efficiency of DIRAC presented here is rather conservative.

### Application on general NP-hard problems

It has been shown that many NP-hard problems, including all of Karp’s 21 NP-complete problems (such as the max-cut problem, 3-SAT problem and the graph coloring problem), have Ising formulations^11^. Hence, we anticipate that DIRAC (with some modifications) could help us solve a wide range of NP-hard problems that have Ising spin glass formulations. We emphasize that to make the current DIRAC framework fully compatible with more complex Ising formulations is nontrivial. In the EA spin glass model, we only have pair-wise or two-body interactions, which can be represented by “edges” connecting spins. When the Ising formulation involves *k*-body interactions (with *k* > 2), we have to leverage the notion of hypergraph and replace the “edge feature” in DIRAC with the “hyperedge feature”^51^, which have been heavily studied in the field of hypergraph and hypergraph learning^52–54^.

We emphasize that GT can be applied to any optimization problem (such as *k*-SAT^55^ and graph coloring^11^) with an Ising formulation. Consider a general Ising formulation:

6 $${{{{{{{\mathcal{H}}}}}}}}=-\mathop{\sum }\limits_{a=1}^{M}{J}_{a}{\sigma }_{{a}_{1}}{\sigma }_{{a}_{2}}\cdots {\sigma }_{{a}_{{k}_{a}}}.$$

Here we have *M* interactions, and the *a*-th interaction involves *k*_*a*_ ⩾ 1 Ising spins (*k*_*a*_ = 1 corresponds to the external field, *k*_*a*_ = 2 corresponds to the two-body interaction we considered in this paper). Note that GT still works (with *t*_*i*_ = ± 1):

7 $$\left\{\begin{array}{lr}{J}_{a}\to {J}_{a}^{{\prime} }={J}_{a}{t}_{{a}_{1}}{t}_{{a}_{2}}\cdots {t}_{{a}_{{k}_{a}}}&\\ {\sigma }_{i}\to {\sigma }_{i}^{{\prime} }={\sigma }_{i}{t}_{i} &\\ \end{array}\right.$$

So that the Hamiltonian/energy remains the same:

8 $${{{{{{{\mathcal{H}}}}}}}}\to {{{{{{{{\mathcal{H}}}}}}}}}^{{\prime} }=-\mathop{\sum }\limits_{a=1}^{M}{J}_{a}{\sigma }_{{a}_{1}}{\sigma }_{{a}_{2}}\cdots {\sigma }_{{a}_{{k}_{a}}}{t}_{{a}_{1}}^{2}{t}_{{a}_{2}}^{2}\cdots {t}_{{a}_{{k}_{a}}}^{2}=-\mathop{\sum }\limits_{a=1}^{M}{J}_{a}{\sigma }_{{a}_{1}}{\sigma }_{{a}_{2}}\cdots {\sigma }_{{a}_{{k}_{a}}}={{{{{{{\mathcal{H}}}}}}}}.$$

As a concrete example, we applied DIRAC to explicitly calculate the max-cut problem (SI Sec. II), a canonical example of the mapping between Ising spin glasses and NP-hard problems^11^. The results are shown in SI Fig. S9. We found that DIRAC consistently outperforms other competing max-cut solvers.

### Interpreting the superior performance of DIRAC

**Fig. 6:**
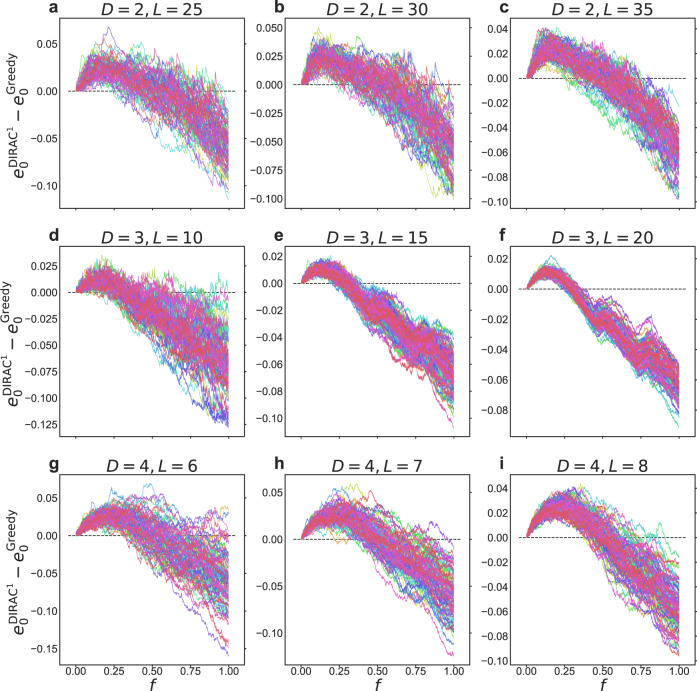
A long-sighted greediness. We compared the energy density difference between DIRAC^1^ and Greedy (${e}_{0}^{{{{{{{{\rm{DIRAC}}}}}}}}}-{e}_{0}^{{{{{{{{\rm{Greedy}}}}}}}}}$) at each step. Here *e*_0_ denotes the disorder averaged “ground-state” energy per spin (predicted by each method), and *f* denotes the fraction of spins that have been flipped. Note that the number of greedy steps of two methods may not be exactly the same, we fill the length of the shorter sequence with its last value, such that the values of these two sequences can be compared one by one. Note that to compare with Greedy step by step more precisely, the results of DIRAC^1^ are achieved by flipping only one spin each step. By contrast, the results of DIRAC^1^ in Fig. 5 are achieved by flipping a fraction of top 1% highest-*Q*-value spins at each step. (**a**–**i**) illustrate the energy gaps for different dimensions and different sides. For each size, we tested using 100 randomly generated EA spin glass instances (with couplings sampled from Gaussian distribution ${{{{{{{\mathcal{N}}}}}}}}(0,1)$), which were represented by 100 curves of different colors. It can be clearly seen that DIRAC^1^ always goes through a high-energy state temporarily in the early stage of the greedy process, so as to reach a much lower energy state in the long run.

In this section, we offer a systematic interpretation on the superior performances of DIRAC. In Fig. 6, we compare the system’s energy difference between DIRAC^1^ and the Greedy algorithm at each greedy step. Note that both methods are performed greedily, the difference is that the former greedily flips the highest-*Q*-value spin at each step while the latter greedily flips the highest-energy-drop spin at each step. (Note that, for a fair comparison, here in DIRAC^1^ at just step we just flip the spin with the highest *Q*-value, instead of flipping a fraction of spins with highest *Q*-values.) The (energy-based) Greedy algorithm represents an extremely short-sighted strategy, since it focuses only on each step’s maximal energy drop. Fig. 6 clearly shows that compared to this short-sighted strategy, DIRAC^1^ always goes through a high-energy state temporarily for the early steps, so as to reach a much lower energy state in the long run. This result implies that DIRAC has learned to make short-term sacrifices for long-term gains. In other words, DIRAC has been trained to be mindful of its long-term goals.

In Fig. S10, we demonstrate that, during each iteration of DIRAC^*m*^ (which is a sequential running of *m* iterations of DIRAC^1^ connected by GTs), there are two interesting phenomena: (1) the fraction of anti-ferromagnetic bonds in the gauge transformed instances keeps decreasing (Fig. S10k); and (2) the *Q*-value distribution becomes more homogeneous (Fig. S10l). In Fig. S3, we show clear evidence that DIRAC^*m*^ significantly outperforms *m* independent DIRAC^1^ (where each DIRAC^1^ is dealing with a random instance with ≈ 50% anti-ferromagnetic bonds). These results implies that the superior performance of DIRAC^*m*^ is related to the decreasing fraction of anti-ferromagnetic bonds and a more homogeneous *Q*-value distribution due to the sequential GTs.

**Fig. 7:**
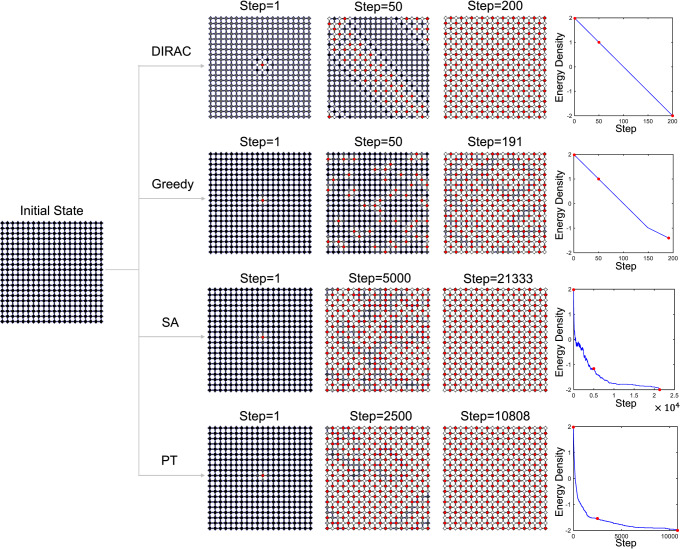
DIRAC attempts to mimic human intelligence in finding the ground state of the anti-ferromagnetic Ising model where all couplings *J*_*ij*_ = −1. Here we demonstrated how different methods, namely DIRAC, Greedy, SA, and PT, reach the ground state in the case of anti-ferromagnetic Ising model where all couplings *J*_*ij*_ = −1. The PT snapshots and energy trend shown here were chosen from the lowest-temperature replica. Also, for SA, here we set the number of sweeps *N*_s_ = 1 (instead of 50) because for SA finding the ground state of the anti-ferromagnetic Ising model is a relatively easy task w.r.t finding the spin glass ground state. All the different methods started from a uniform initial state (the leftmost snapshot in the figure), where all the spin values are set to be +1 (black). In the following snapshots, red sites represent sites with spin value − 1. The grayscale value was determined as follows: For DIRAC, all the *Q*-values were rescaled to the range from 0 (the smallest *Q*-value, white) to 1(the largest *Q*-value, black); For Greedy, SA and PT, the site energies ranging from − 4 to + 4 are rescaled to the range from 0 (site-energy = − 4, white) to 1 (site-energy = + 4, black) and take the power of four for clearer visualization. In any case, a darker site is always more likely to be flipped in the next step than a lighter site. In the rightmost column, the trend of the energy density drop is shown. Here the step is defined as each try to flip a spin, so in a given step of SA or PT, the spin is not necessarily flipped, if the try is not accepted in the Metropolis-Hastings procedure. We can see that for the anti-ferromagnetic Ising model, DIRAC demonstrates a remarkable long-sighted vision by making a smart move every time (so that its spin configuration displays a clear checkerboard pattern), implying that it seeks to mimic human intelligence in solving the ground state problem. In supplementary movies, we showed the complete process of how these methods execute on this simple anti-ferromagnetic Ising model.

The results shown in Fig. S3 and Fig. S10 prompt us to ask if the superior performance of DIRAC over other methods can be better visualized in an extreme case, i.e., an anti-ferromagnetic Ising model where *J*_*ij*_ = − 1 for all the bonds. It is well known that this simple model has a ground state with a checkerboard pattern in the spin configuration (as shown in Fig. 7, first row, step = 200, where the red/white sites represent −1/+1 spins, respectively). However, for classical energy-based heuristic algorithms (e.g., Greedy, SA and PT), this ground state cannot be found easily. Consider an anti-ferromagnetic Ising model on a 20 × 20 square lattice with periodic boundary conditions. The last column in Fig. 7 shows the trend of energy vs. the number of steps taken by those heuristic algorithms. For the Greedy algorithm, it ran for 191 steps and got stuck in a local minimum whose energy is significantly higher than the ground state energy. For SA or PT (the coldest replica), it took 21,333 or 10,808 steps to finally reach the ground state, respectively. By contrast, DIRAC only took 200 = 20 × 20/2 steps (i.e., flipping exactly half of the spins) to reach the ground state for this lattice. In other words, DIRAC did not make any wrong decision in the whole process, which is remarkable.

To further explain why DIRAC is so “smart” in this case, we look at the snapshots shown in Fig. 7. All the different algorithms start from a uniform initial state where all the spin values are set to be +1. Note that in the snapshots, red sites represent spin values − 1. Sites with grayscale colors represent spin values + 1, and the grayscale of each site is determined by its *Q*-value or site-energy. For DIRAC, a darker site corresponds to a higher *Q*-value; for Greedy, SA and PT, a darker site corresponds to a higher site-energy. All the algorithms always tend to flip a darker site with a higher *Q*-value or site-energy. But DIRAC differs from other algorithms in the following way. Since the instance composes of purely anti-ferromagnetic bonds, the *Q*-values of different nodes (spins) are all the same at the beginning. After the first spin is flipped (see the center node in the step = 1 snapshot of DIRAC in Fig. 7), there are two consequences: (1) all its first nearest neighbors’ *Q*-values are “smartly” decreased, rendering them less likely to be flipped in the future; (2) all its second nearest neighbors’ *Q*-values are “smartly” increased, rendering them more likely to be flipped in the next step. In a sense, DIRAC has a long-sighted vision that cleverly leverages the nature of a purely anti-ferromagnetic Ising model. As a result, the intermediate snapshots (e.g., in step = 50) display a clear “stripe” pattern that “grows” from the first flipped spin. By contrast, other algorithms are short-sighted, try almost random flips at the beginning, then make incorrect flips and get stuck in the local minimum. The Greedy algorithm got stuck in a local minimum forever. SA and PT can jump out of their local minimum, but it took them very long time to achieve the final ground state.

Taken together, DIRAC seeks to mimic human intelligence in solving the ground state problem. For the spin glass ground state problem, it learns to scarify short-term satisfaction for long-term gains. For the anti-ferromagnetic Ising model, it demonstrates a remarkable long-sighted vision by making a smart move every time.

## Discussion

This work reports an effective and efficient deep RL-based algorithm, DIRAC, that can directly calculate the ground states of EA spin glasses. Extensive numerical calculations demonstrate that DIRAC outperforms state-of-the-art algorithms in terms of both solution quality and running time. Besides, we also evaluate DIRAC’s superior performances under different scenarios, e.g., different coupling distributions (Gaussion vs. Bimodal vs. Uniform) (Fig. S11); different topological structures (trees vs. loopy trees vs. lattices) (Fig. S12); different hardness regimes (Fig. S13, Fig. S14) and different spin glass models (EA vs. Sherrington-Kirkpatrick) (Fig. S15), see SI Sec. III for more details. Through a pure data-driven way and without any domain-specific guidance, DIRAC smartly mimics the human intelligence in solving the spin glass ground state problem. In particular, DIRAC enables a much faster finding of ground states than existing algorithms, and it can greatly improve annealing-based methods (e.g., SA and PT) to reach the lowest system energy for all dimensions and sides.

Note that in our implementations of annealing-based methods (e.g., SA and PT), we took the parameters of SA and PT from Ref. ^50^ and Ref. ^49^. We found that our implementations of SA and PT were able to generate similar results as (or, arguably, a slightly better performance than) what were reported in existing works (see SI, Fig. S16 and Fig. S17). We emphasize that even if SA or PT itself can be further improved, we can still use DIRAC as a plug-in to enhance the improved version of SA or PT. Hence, we are not only interested in comparing DIRAC with the state-of-the-art implementation of SA or PT, but also interested in comparing DIRAC-enhanced thermal annealing algorithms with their corresponding vanilla algorithms (as shown in SI Fig. S5).

In the future, advances in deep graph representations may enable us design a better encoder, and developments of RL techniques may help a more efficient training. Both would further improve DIRAC’s performances to find the ground states of Ising spin glasses. The utilization of GT in DIRAC and the way of combining DIRAC and annealing-based methods may also inspire many other physics-guided AI research. Our current framework is just the beginning of such a promising adventure.

## Methods

### DIRAC

For DIRAC, Tab. S2 lists the values of its hyper-parameters, which were determined by an informal grid search. We only tried to tune a few hyper-parameters, including the discount factor *γ*, delay reward steps *n* and the message-passing steps *K*. The results are shown in Fig. S18, Fig. S19, and Fig. S20. Therefore DIRAC’s performances can be further improved by a more systematic grid search. For example, in Fig. S20, we found that the agent trained using the same *K* value as that in testing often yields the best performance, and this observation stands for different system sizes. This contradicts our intuition that a larger *K* should always obtain better performances on large systems due to a better capture of the long-range correlations. We suspect that this may be due to the inconsistency between *K* and the embedding dimension *d* (i.e., the size of node embedding vector, which is always set to be *d* = 64 in all our calculations). We anticipate that *d* should be higher for higher *K* so that longer correlations can be encoded in the node embedding vector. Systematically testing this idea is beyond the scope of the current study. For more implementation details, please see SI Sec. I.

### SA

For SA, we linearly annealed the temperature from a high value to a low one, the number of temperatures is set to be *N*_t_. For each temperature, we performed *N*_s_ sweeps of explorations, each sweep contains *N* (number of spins) random moves. The values of hyper-parameters are determined from Ref. ^50^, i.e., setting the maximal inverse temperature ${\beta }_{\max }=5$ and the minimal inverse temperature ${\beta }_{\min }=0$. We set *N*_t_ = 100, which is consistent with the first row in TABLE I in Ref. ^50^. Ref. ^50^ also pointed out that the optimized value of *N*_t_ × *N*_s_ should be around 5000. In our case, we set *N*_s_ = 50 and *N*_t_ = 100. For more implementation details, please see SI Sec. IV.

### PT

For PT, we chose *N*_r_ = 20 replicas, whose temperatures range from 0.1 to 1.6 with equal interval^49^, initialized with random configurations. Within each epoch, we attempted random flips for *N* (the number of spins) times. After these random flips, we randomly picked up two replicas and exchanged their spin configurations. The lowest energy and the corresponding spin configuration of all the replicas were recorded during the whole process. For more implementation details, please see SI Sec. IV.

### Supplementary information

Supplementary Information

Peer Review File

Description of additional Supplementary File

Supplementary Movie 1

Supplementary Movie 2

Supplementary Movie 3

Supplementary Movie 4

## Data Availability

The data used to reproduce the results in this paper are publicly available through Zenodo^56^ (10.5281/zenodo.7562380).

## Code availability

The source code of DIRAC (and its variants), as well as the two baseline methods, SA and PT, are publicly available through Zenodo^56^ (10.5281/zenodo.7562380or on GitHub (https://github.com/FFrankyy/DIRAC.git).
