# Supplementary material for: Searching for spin glass ground states through deep reinforcement learning
Source: Nat Commun. 2023 Feb 9;14:725. doi: 10.1038/s41467-023-36363-w (PMC9911406; doi:10.1038/s41467-023-36363-w)
Supplement: Supplementary file 1 — Supplementary Information [file 41467_2023_36363_MOESM1_ESM.pdf]

Supplementary Information for
Searching for spin glass ground states through deep reinforcement learning

Changjun Fan,[1,*] Mutian Shen,[2,*] Zohar Nussinov,[2,3]
Zhong Liu,[1] Yizhou Sun,[4,†] and Yang-Yu Liu[5,6,‡]

[1] *College of Systems Engineering,*
National University of Defense Technology, Changsha, 410073, China

[2] *Department of Physics,*
Washington University in St. Louis, Campus Box 1105,
1 Brookings Drive, St. Louis, MO 63130, USA

[3] *Rudolf Peierls Centre for Theoretical Physics,*
University of Oxford, Oxford OX1 3PU, United Kingdom

[4] *Department of Computer Science,*
University of California, Los Angeles, CA, 90024, USA

[5] *Channing Division of Network Medicine,*
Brigham and Women's Hospital and Harvard Medical School, Boston, MA, 02115, USA

[6] *Center for Artificial Intelligence and Modeling,*
The Carl R. Woese Institute for Genomic Biology,
University of Illinois at Urbana-Champaign, Champaign, IL 61820, USA

(Dated: January 23, 2023)

* These authors contributed equally to this work

† Author to whom correspondence should be addressed. Email: yzsun@cs.ucla.edu

‡ Author to whom correspondence should be addressed. Email: yyl@channing.harvard.edu

CONTENTS

List of Figures 4

List of Tables 5

List of Algorithms 5

I. More details of DIRAC 6
- A. Encoding 6
- B. Decoding 8
- C. Offline training 8
- D. Online application 10
- E. Time complexity 11
- F. Ablation studies on SGNN 13
- G. Training using different coupling distributions 14
- H. Use DIRAC as a plug-in in Monte-Carlo Methods 14

II. Apply DIRAC to solve the max-cut problem 16
- A. Ising spin glass formulation of the max-cut problem 16
- B. Results 17

III. DIRAC's superior performances over other methods under different scenarios 18
- A. Different topological structures 18
- B. Different hardness regimes 18
- C. Different spin glass models 20

IV. Existing methods for finding ground states of Ising spin glasses 20
- A. Exact algorithms 20
  - 1. Branch and Bound 21
  - 2. Minimum Weight Perfect Matching 21
- B. Heuristic algorithms 21
  - 1. Simulated Annealing (SA) 22
  - 2. Population Annealing (PA) 22
  - 3. Parallel Tempering (PT) 22

C. Machine learning algorithms 24

Supplementary References 58

References 58

LIST OF FIGURES

S1 Illustration of two key steps in each per-layer update (Eq. 1) 32
S2 Ablation study for SGNN 33
S3 DIRAC^m performs significantly better than m independent DIRAC^1 34
S4 Comparison of SA, PT and their DIRAC-enhanced versions: DIRAC-SA, DIRAC-PT. 35
S5 Statistical significance test of the main text Fig. 5 37
S6 Effects of batch-nodes-selection on DIRAC's performances 38
S7 Number of DIRAC^m iterations scales linearly with $\log N$ 39
S8 DIRAC's training convergence 40
S9 DIRAC's application on the max-cut problem 41
S10 An illustration of the workflow of DIRAC^m 42
S11 DIRAC's performances on different testing coupling distributions using different training coupling distributions 43
S12 Comparing the performance of DIRAC and Belief Propagation in solving the ground state problem of the spin glasses on different topological structures . . 44
S13 Three types of sub-lattices to "plant" a spin glass instance with desired hardness 45
S14 Comparing the performance of DIRAC and baseline methods in solving the ground state of spin glasses problem with different levels of hardness 46
S15 Comparing the performance of DIRAC and PT in solving the ground state of the Sherrington-Kirkpatrick (SK) spin-glass model 48
S16 Our SA implementation can reproduce results reported in the literature 50
S17 Our PT implementations can reproduce results reported in the literature 51
S18 Impact of discount factor γ on DIRAC's performance 52
S19 Impact of the delay reward parameter n on DIRAC's performance 53

S20 Training DIRAC using the same number of message passing steps as that in the application instances yields the best performance 54

LIST OF TABLES

S1 Time complexity analysis 55
S2 List of hyper-parameters, their values and brief descriptions 56

LIST OF ALGORITHMS

S1 Parallel Tempering (PT) 25
S2 Simulated Annealing (SA) 26
S3 SGNN encoder 27
S4 Training process of DIRAC 28
S5 PlayGame(ϵ) 29
S6 DIRAC-PT 30
S7 DIRAC-SA 31

I. MORE DETAILS OF DIRAC

In this section, we introduce more details about DIRAC's encoding, decoding, offline training and online application, and analyze its time complexity in both training and application. Finally, we conduct ablation studies for DIRAC's encoder SGNN to reveal key components of SGNN that contribute to its success.

A. Encoding

We seek to represent each spin using an embedding vector of size d. This vector should capture both the spin's features and its long-range correlations with other spins. Coupling strengths between spins, i.e., J_{ij}, which are crucial to determine the spin glass ground states, should also be encoded in this vector. Since a hypercubic lattice can be regarded as a special graph, we consider using the graph neural network (GNN) [1–3] as the encoder. The encoded representations can be learned in a task-specific way, and thus are superior to those hand-crafted features that are ad hoc and less representative.

However, as we discussed in the main text, most current GNN architectures designed for general graphs cannot be applied directly to hypercubic lattices. As a result, we designed SGNN to better encode the spin glass instances on hypercubic lattices. Specifically, SGNN follows two steps of neighbor-aggregation (i.e., edge-feature aggregation and node-feature aggregation) at each layer to incorporate both edge features and node features. Specifically, a per-layer update of a SGNN encoder involves the following two key steps (see Fig. S1):

$$\begin{cases} \mathbf{h}_{ij}^{(l)} &= \mathrm{ReLU}([\mathbf{W}_3(\mathbf{h}_i^{(l-1)} + \mathbf{h}_j^{(l-1)}), \mathbf{W}_4\mathbf{h}_{ij}^{(0)}]), \\ \mathbf{h}_i^{(l)} &= \mathrm{ReLU}([\mathbf{W}_5 \sum_{v_j \in \mathcal{N}(v_i)} \mathbf{h}_{ij}^{(l)}, \mathbf{W}_6\mathbf{h}_i^{(l-1)}]). \end{cases} \tag{1}$$

Here $\mathbf{h}_{ij}^{(0)} = \mathrm{ReLU}(\mathbf{W}_2\mathbf{x}_{ij}) \in \mathbb{R}^d$, $\mathbf{h}_i^{(0)} = \mathrm{ReLU}(\mathbf{W}_1\mathbf{x}_i) \in \mathbb{R}^d$ are initial embedding vectors for edge e_{ij} and node v_i, respectively. $\mathbf{x}_{ij} \in \mathbb{R}^4$ and $\mathbf{x}_i \in \mathbb{R}^D$ are the edge input feature vector and the node input feature vector, respectively. $\mathbf{W}_1 \in \mathbb{R}^{d\times D}$, $\mathbf{W}_2 \in \mathbb{R}^{d\times 4}$, $\mathbf{W}_3, \mathbf{W}_4, \mathbf{W}_5, \mathbf{W}_6 \in \mathbb{R}^{\frac{d}{2}\times d}$ are all learnable encoding parameters. $[\cdot]$ denotes concatenation.

The first equation in Eq. 1 computes a message $\mathbf{h}_{ij}^{(l)}$ for each edge e_{ij} at layer l, which combines the edge's initial representation $\mathbf{h}_{ij}^{(0)}$ and its adjacent nodes' representations $\mathbf{h}_i^{(l-1)}$

and $\mathbf{h}_j^{(l-1)}$ in the previous layer. The second equation calculates a message $\mathbf{h}_i^{(l)}$ for each node v_i at layer l, which combines its representation from the previous layer $\mathbf{h}_i^{(l-1)}$ and the aggregated messages from its neighborhood $\{\mathbf{h}_{ij}^{(l)}\}_{v_j \in \mathcal{N}(v_i)}$.

The edge input feature is set as $\mathbf{x}_{ij} = (J_{ij}, \sigma_i, \sigma_i\sigma_j, 1)^\top \in \mathbb{R}^4$ for each edge e_{ij}, where σ_i denotes the spin variable (either $+1$ or -1), J_{ij} denotes the coupling strength between spin i and spin j, the constant 1 is commonly used in practice as a bias [4]. Note that the edge feature is independent of side L and dimension D. For the node input feature in a D-dimensional hypercubic lattice with periodic boundary conditions, since there are no node attributes and useful node structure statistics (all nodes share the same degree $2D$), we here establish a rectangular coordinate system with an arbitrary node in the lattice chosen as the origin. Then each node's coordinate is set as its input feature, i.e., $\mathbf{x}_i = (x_i^{(1)}, \cdots, x_i^{(D)})^\top \in \mathbb{R}^D$. Hence, the size of the node feature vector equals the system dimension D, and is independent of L. As a result, SGNN allows the same encoder to accept inputs from systems of different sizes, but not different dimensions. In other words, DIRAC trained on D-dimensional hypercubic lattices of small side could generalize to D-dimensional hypercubic lattices of large side, but DIRAC trained on $D = 2$ systems could not generalize to $D = 3$ or $D = 4$ systems.

Popular aggregation methods include mean/max pooling [3], sum pooling [4], attention pooling [5], etc. We also conduct residual connections between consecutive layers (Eq. 2) to alleviate the "over-smoothing" problem [1], i.e., we set.

$$\mathbf{h}_i^{(l)} = \mathbf{W}_7[\mathbf{h}_i^{(l)}, \mathbf{h}_i^{(l-1)}], \tag{2}$$

where $\mathbf{W}_7 \in \mathbb{R}^{d \times 2d}$, and $[\cdot]$ denotes concatenation.

We repeat the above computations for K layers to obtain each node or spin's embedding vector. The final embedding for node v_i after K layers is $\mathbf{z}_i = \mathbf{h}_i^{(K)} \in \mathbb{R}^d$, which captures both the node's position and its long-range couplings with neighbors within K hops. To capture the state s of the whole spin glass instance, the summation over all nodes, $\mathbf{z}_s = \sum_{i=1}^N \mathbf{z}_i$ is a straightforward way of aggregating the whole system, which is invariant to the permutation of nodes ordering. There are also more complex graph-level pooling methods, including the virtual node surrogate [6], DiffPool [7], etc., with which we can utilize for the state embedding. Algo. S3 describes more details of SGNN.

B. Decoding

The decoding part maps the state-action pair $(s, a^{(i)})$ to a scalar $Q(s, a^{(i)}; \Theta)$, which predicts the expected future cumulative rewards if taking action $a^{(i)}$ under state s, and following the policy $\pi_\Theta(a^{(i)}|s)$ till the end of the episode (i.e., till all the spins have been flipped). We parameterize the Q function with a classical two-layer MLP with ReLU activation, i.e.,

$$Q(s, a^{(i)}; \Theta) = \text{MLP}(\mathbf{z}_s, \mathbf{z}_i; \Theta) = \mathbf{W}_{10}\text{ReLU}(\mathbf{W}_9\text{ReLU}(\mathbf{W}_8[\mathbf{z}_s, \mathbf{z}_i])). \tag{3}$$

Here $\mathbf{W}_8 \in \mathbb{R}^{d\times 2d}$, $\mathbf{W}_9 \in \mathbb{R}^{\frac{d}{2}\times d}$, and $\mathbf{W}_{10} \in \mathbb{R}^{1\times \frac{d}{2}}$ are learnable weight parameters. $\mathbf{z}_s \in \mathbb{R}^d$ and $\mathbf{z}_i \in \mathbb{R}^d$ are the embedding vectors calculated from Algo. S3, for state s and action $a^{(i)}$ respectively. Note that we don't actually need to compute the Q-score for each state-action pair using Eq. 3. For more efficient computations, there is a separate output unit for each possible action in our implementations and we only need to feed the state embeddings as the input, and then we can compute Q-values for all possible actions under a given state s with only one single forward pass through the Q-network [8].

C. Offline training

For DIRAC, the parameters of its Q-network $\Theta = \{\Theta_\mathcal{E}, \Theta_\mathcal{D}\}$, $\Theta_\mathcal{E} = \{\mathbf{W}_k\}_{k=1}^{7}$ are the encoding parameters, and $\Theta_\mathcal{D} = \{\mathbf{W}_k\}_{k=8}^{10}$ are the decoding parameters. We have predicted the Q-values for each state-action pair $(s, a^{(i)})$ using the Q-network. If given the "true labels" $Q_{\text{true}}(s, a^{(i)})$ at hand, we can directly update parameters with the end-to-end gradient descents on minimizing the gap between these two values. However, in practice, we do not have these labels. As such, we have to use reinforcement learning (RL) to deal with this "delayed label" case, in which the labels to judge whether the predictions are correct or not can be obtained only in the end of an episode, and there are no such labels available before the end of an episode.

There are various off-the-shelf RL algorithms. Here we choose Q-learning, for its higher sample efficiency than their policy gradient counterparts [2, 9]. Besides, the learned Q-value could also provide an action score that helps us better understand the policy's behavior.

We now briefly introduce Q-learning. Suppose $\pi_\Theta(a_t|s_t)$ is defined as the policy denoting the probability the agent takes action a_t at time t under state s_t, and our training goal is

to learn an optimal Q-value function $Q(s_t, a_t; \Theta)$, which could estimate the expected future cumulative rewards if the agent takes action a_t at time t under state s_t and then follows the policy π_Θ for the remaining episode.

We define some terms to better describe the training process. An episode is the entire ground states finding process, which starts from the all-spins-up configuration and ends at the all-spins-down configuration. A trajectory produced from an episode corresponds to a state-action-reward sequence $(s_0, a_0, r_0, s_1, a_1, r_1, s_2, ..., s_\mathrm{T})$, where s_T denotes the terminal state. To play one game is to finish an entire episode on the lattice, including generating the trajectory, collecting the experience transitions $(s_t, a_t, r_{t,t+n}, s_{t+n})$, where $r_{t,t+n} = \sum_{k=0}^{n-1} \gamma^k r(s_{t+k}, a_{t+k}, s_{t+k+1})$, and storing them into the experience replay buffer $\mathcal{B}$ (which is a queue that maintains S_buffer most recent experience transitions). Here, the delay reward parameter n, the discount factor γ, and the buffer size S_buffer are all hyper-parameters. For each episode, the agent adopts the ϵ-greedy strategy, which takes the highest-Q action with probability 1-ϵ, and takes random actions otherwise. In our calculations, ϵ is linearly annealed from $\epsilon_\mathrm{initial}$ to ϵ_final over n_ϵ episodes to balance exploration and exploitation [10]. Large ϵ tends to favor random actions and encourages the exploration of the search space, and small ϵ tends to facilitate the exploitation of the learned Q-values. In our calculations, we set $n = 5$, $\gamma = 1$, $S_\mathrm{buffer} = 5 \times 10^4$, $\epsilon_\mathrm{initial} = 1$, $\epsilon_\mathrm{final} = 5 \times 10^{-2}$ and $n_\epsilon = 5 \times 10^4$.

We train the DIRAC agent by playing large amounts of games on randomly generated EA instances (until the training process converges, Fig. S8). Every C_1 episodes, we generate 1,000 random instance with couplings drawn from a Gaussian distribution with zero mean and unit variance. Alongside the game, we use the experience replay buffer $\mathcal{B}$ to store S_buffer most recent experience transitions. Meanwhile, to update parameters Θ, we randomly sample mini-batch transitions from $\mathcal{B}$, and perform Adam gradient descents [11] with learning rate $\alpha = 10^{-4}$ to minimize the following loss function:

$$\mathcal{L} = \mathbb{E}_{(s_t, a_t, r_{t,t+n}, s_{t+n}) \sim \mathcal{B}} \left[\left(r_{t,t+n} + \gamma \max_{a_{t+n}} Q(s_{t+n}, a_{t+n}; \hat{\Theta}) - Q(s_t, a_t; \Theta) \right)^2 \right], \quad (4)$$

where the delay reward parameter n determines the number of steps we wait before updating parameters Θ, so as to collect a more accurate estimate of the future rewards. In our calculations, we usually set $n = 5$. The discount factor γ determines the importance of future rewards. A discount factor of 0 will make the agent short-sighted by only considering immediate rewards, while a factor approaching 1 will make it strive for a long-term high

reward. If $\gamma > 1$, the action values may diverge. In our calculations, we usually set $\gamma = 1$. $\hat{\Theta}$ is the target parameter set, which will only be updated with Θ every C_3 episodes, and is fixed during the individual updates. Techniques of the experience replay buffer and target network enable a more stable training process [8].

Every C_2 episodes, we evaluate the model using the validation data, i.e., 100 randomly generated instances from the same coupling distribution and with the same size as the training instances. The training size is set to be $L \leq 15$ for 2D instances, $L \leq 10$ for 3D instances, and $L \leq 6$ for 4D instances. The validation performance is measured by the approximation ratio (e_0/e_1, e_0 is the energy density computed by DIRAC, e_1 is calculated by the Greedy algorithm). Note that C_1, C_2, and C_3 are hyper-parameters. In our calculations, we set $C_1 = 5,000$, $C_2 = 300$ and $C_3 = 1,000$.

During training, we take an implicit "stop" action at each decision step, i.e., clip the value of $\max_{a_{t+n}} Q(s_{t+n}, a_{t+n}; \hat{\Theta})$ in Eq. 4, and make sure it is positive throughout the episode. This is because we should stop the decision steps when the predicted future returns are negative (indicating no further gains since then). We believe this is important to make the Q-learning correct in our case. Algo. S4 describes the whole training procedure in detail.

In Algo. S4, Gen_Training_data() and Gen_Validation_data() refer to the generation of training instances and validation instances, Test() refers to evaluate the model training at the current stage.

D. Online application

In application, DIRAC adopts the Q-greedy strategy, which starts from the all-spins-up configuration $\{\sigma_i = +1\}$ for an input instance, and flips the highest-Q spin step by step till the all-spins-down configuration $\{\sigma_i = -1\}$. The spin configuration of the lowest system energy encountered during the process is returned as the predicted ground state. This kind of finite-horizon MDP helps DIRAC pick the right move without much regretting, besides, always starting from the same uniform configuration helps reduce the potential trajectory space and thus requires less data for training. By contrast, an example of infinite-horizon MDP can be modeled as following: the agent, starting from any spin configuration, keeps flipping spins until the system reaches a local minimum, which does not necessarily coincide with the global minimum (i.e., the ground state). In our experiments, we found the infinite-

horizon MDP model would cause the training process to diverge, and lead to poor application performance. There are also other finite-horizon MDPs, for example, the agent starts from any configuration, and keeps flipping spins (repeated flips are allows) until a fixed number of steps (such as kN steps with $k = 1, 2, ...$). However, none of them empirically worked better than current DIRAC's MDP design. In this work, we adopt the technique of gauge transformation (GT) [12] which is able to switch the spin glass system between any two configurations while keeping the system energy invariant. In this way, DIRAC is able to handle any random input spin configuration.

During application, we test the well-trained agent on larger instances ($L = [15, 20, 25]$ for $D = 2$, $L = [10, 15, 20]$ for $D = 3$, and $L = [6, 7, 8]$ for $D = 4$). For each size, we generate 50 random instances. To speed up the test speed, we utilize a batch nodes selection strategy [6], which flip a small fraction of highest-Q spins at each adaptive step till the end. This differs from the one-by-one flip in training. We found that if we flipped around top 1% of spins (based on their Q-values) at each step, we were able to approach the one-by-one flip performance without much accuracy loss while obtained a significant computational speed-up (Fig. S6).

E. Time complexity

We analyze DIRAC's time complexity in each phase.

Offline training. During the offline training phase, we train DIRAC for a total $\Omega = 10^6$ iterations (or episodes). For each iteration, its time complexity is $O(N)$ (see DIRAC[1] in the application phase time complexity analysis section), and the whole training complexity is proportional to the number of training iterations that are needed to converge, which is hard to be theoretically analyzed. However, we observe in Fig. S8 that DIRAC always converge very quickly in a short period. For example, DIRAC took about 30 seconds every 300 training iterations on 3D Ising spin glass instances, which was about 28 hours for a total one million iterations (all the calculations were conducted on a 20-core computer server with 512GB memory and a 16GB Tesla V100 GPU). Meanwhile, it actually converged in the middle, taking an estimated 90,000 iterations (about 2.5 hours). Notably, the training phase only needs to be performed once for each dimension of the hypercubic lattice, and then we can utilize the trained agent infinite times in the application phase for large instances at

the same dimension.

Online application. During the online application phase, we have two basic strategies: DIRAC^1 and DIRAC^m. The time complexity of DIRAC^1 is determined by three parts. The first part is from encoding. As shown in Algo. S3, the running time of SGNN can be estimated as $K(M + \langle k \rangle N)$, where the hyper-parameter K is the number of propagation steps, and is usually a small constant (we set $K = 5$ in our calculations), $\langle k \rangle = 2D$ is the mean degree of nodes in the D-dimensional hypercubic lattice, M is the number of edges (for lattice structures with periodic boundary conditions, we have $M = \frac{\langle k \rangle N}{2} = ND$), N is the number of nodes (spins). Consequently, DIRAC^1's encoding time is proportional to $3KND$. The second part is from the decoding step. Once the instance has been encoded, we need to compute Q-values for all spins with time complexity $O(N)$. The last part comes from the greedy flip step, in which we flip a finite fraction f of spins with the highest Q-values at each step. Thus the total number of greedy steps is $\lceil \frac{1}{f} \rceil$ ($\lceil x \rceil$ is the ceiling function), which is a constant number with regard to the system size. Taken together, the total application time for DIRAC^1 can be estimated as $\lceil \frac{1}{f} \rceil \times (3KND + N)$, rendering a time complexity of $O(N)$. DIRAC^m essentially repeats m rounds of DIRAC^1, thus its time complexity is given by $O(mN)$. Numerical simulations suggest that m scales linearly with $\log N$ (Fig. S7). Hence the time complexity of DIRAC^m is estimated as $O(N \log N)$.

Comparison with other baseline methods. We compare the time complexity of DIRAC with that of other baseline methods in Tab. S1.

The Greedy algorithm keeps flipping those largest-energy-drop spins until the system energy converges. It is very challenging, if not impossible, to determine the time complexity of the Greedy algorithm, because it terminates only when the energy converges. For simplicity and without the loss of competition fairness to DIRAC, here we consider the best scenario $O(N)$ as the Greedy algorithm's time complexity.

For SA, we linearly annealed the temperature from a high value to a low one, the number of temperatures is set to be N_{t}. For each temperature, we performed N_{s} sweeps of explorations, each sweep contains N random moves. Therefore the total time complexity of SA is $O(NN_{\mathrm{t}}N_{\mathrm{s}})$.

For PT, we chose $N_{\mathrm{r}} = 20$ replicas, whose temperatures range from 0.1 to 1.6 with equal interval [13], initialized with random configurations. Within each epoch, we attempted random flips for N times. After these random flips, we randomly picked up two replicas

and exchanged their spin configurations. The lowest energy and the corresponding spin configuration of all the replicas were recorded during the whole process. As such, for a total number of N_e epochs, the time complexity of PT is $O(NN_\mathrm{e}N_\mathrm{r})$.

It should be noted that it is more effective to do multiple runs (each run starts from a new initial configuration) for Greedy, SA and DIRAC, and choose the lowest energy from among the runs. We do not consider the number of runs into their time complexity, since these runs all start from independent configurations, and thus can be trivially parallelized. For PT, it explores different configurations by running lots of epochs, however, the starting configurations of these epochs are related, and thus can only be computed sequentially.

F. Ablation studies on SGNN

In this work, our encoder SGNN follows the standard message-passing framework, but is specifically designed to represent the hypercubic lattice structure, which thus leads to several ingredients that are different from most existing GNN architectures designed for general graphs. In this section, we performed ablation studies to see whether they are really needed.

Generally speaking, SGNN's ingredients include node input features, edge input features, and the way of per-layer-update. For node input features, since there are no node attributes and useful node structure statistics (all nodes share the same degree), we use each spin's coordinates (in the rectangular coordinate system that is established by taking an arbitrary spin as the origin) as input features. To see whether this ingredient helps, we designed an ablated agent, named DIRAC_Ablate1, which instead uses the constant one as the node input feature.

For edge input features, we used $(J_{ij}, \sigma_i, \sigma_i\sigma_j, 1)^\top$ as the input feature for each edge e_{ij}. To see whether this ingredient helps, we designed an ablated agent, named DIRAC_Ablate2, which initializes each edge's feature as $(J_{ij}, \sigma_i, \sigma_j, 1)^\top$.

For the way of per-layer-update, traditional GNN architectures [1–3, 5, 14, 15] often focus on only one node-centric update each layer, and the way they deal with edge information is often to treat as a part of node's neighborhood. We here performed two key updates each layer to capture more edge weight information. To see whether this ingredient helps, we designed an ablated agent, named DIRAC_Ablate3, which conducts only one following

node-centric update each layer:

$$\mathbf{h}_i^{(k)} = \mathrm{ReLU}([\mathbf{h}_i^{(k-1)}, \sum_{j\in\mathcal{N}(i)} \mathbf{h}_j^{(k-1)}, \sum_{j\in\mathcal{N}(i)} \mathbf{h}_{ij}^{(0)}]), \tag{5}$$

with $\mathbf{h}_{ij}^{(0)} = \mathbf{x}_{ij} = (J_{ij}, \sigma_i, \sigma_i\sigma_j, 1)^\top$.

We trained all ablated models on small instances and tested them on larger ones, and then compared them with the original DIRAC (termed as DIRAC_Vanilla). As shown in Fig. S2, we found: 1) All ingredients contribute to DIRAC's performances. 2) The edge-centric-update contributes the most.

G. Training using different coupling distributions

The results presented so far are for Gaussian EA spin glass instances, of which the couplings $\{J_{ij}\}$ were sampled from the Gaussian distribution. In Fig. S11, we used Gaussian, Bimodal and Uniform distributions to generate $\{J_{ij}\}$ in training and test instances separately, and analyzed their impacts on DIRAC. We found that DIRAC performs the best when the coupling strengths in the test instances were generated from the same distribution as that in the training instances, and DIRAC trained with instances of Gaussian couplings generalizes better than those trained with instances with coupling strengths sampled from the other two distributions.

H. Use DIRAC as a plug-in in Monte-Carlo Methods

For those annealing-based Monte-Carlo algorithms, such as SA and PT, the key component is the energy greedy procedure, which tends to flip the energy-drop spin flips and also accepts those "bad" (energy-up) flips with a temperature-dependent probability. The higher the temperature is, the higher the acceptance probability of "bad" flips is. In the main-text Fig. 6, we have shown that DIRAC^1 can be considered as another greedy procedure, but with a longer-sighted greediness than the traditional energy-based greedy strategy. Therefore, it's natural to replace the energy-based greedy procedure with DIRAC^1 in these Monte-Carlo methods, and further improve their performances.

The most straightforward way of replacement or plug-in is to apply DIRAC to the final results obtained by these algorithms to see whether DIRAC could further improve their

results. Another simple idea is to replace the energy-greedy procedure with DIRAC only for the lowest-temperature system (SA) or replica (PT), since the system has been very close to the ground states at low temperatures, and more efficient search methods are required here. However, in our practice, we found both ideas are less effective than the following strategy (termed as DIRAC-PT and DIRAC-SA in the main text).

To describe this strategy more clearly, we first define some technical terms. Energy-descent refers to the operation of accepting a move that can lower the system energy. DIRAC-descent refers to the operation of applying DIRAC[1] to reduce the system energy. Energy-based Metropolis Hastings (EMH) (Line 5-11 in Algo. S6) is a procedure that uses the energy-descent operation to lower the system energy and also accepts those "bad" moves ($\Delta E > 0$) with the probability $\min[1, \exp(-\beta\Delta E)]$, where β is the inverse temperature, ΔE is the energy change after the move. DIRAC-based Metropolis Hastings (DMH) (Line 14-18 in Algo. S6) is a procedure that uses the DIRAC-descent operation to lower the energy, and if the system reaches a local minimum (i.e., $\Delta E = 0$), the spin configuration will be perturbed by flipping each spin with probability:

$$P_{\text{randomize}} = q(\beta_{\max} - \beta_t)/(\beta_{\max} - \beta_{\min}) \tag{6}$$

Here $\beta_{\min}$, $\beta_{\max}$, and β_t represent the maximum, minimum and current value of β, respectively, q is a hyper-parameter controlling the perturbation magnitude, and in our calculations we set $q = 0.5$.

This plug-in strategy makes the system (for SA) or replicas (for PT) at each iteration DIRAC-enabled. More specifically, the system or replicas at each iteration could either choose EMH or DMH to lower the system energy (Fig. S4 (b,d)), the decision probability p is a hyper-parameter. In our calculations, we set $p = 0.5$, indicating the two procedures can be chosen with equal chance. See Algo. S6 and Algo. S7 for detailed descriptions of DIRAC-PT and DIRAC-SA. Note that for these DIRAC plug-in strategies, the detailed balance is not satisfied because the algorithms make decisions based on the Q-values, rather than the bond energy.

II. APPLY DIRAC TO SOLVE THE MAX-CUT PROBLEM

The Ising spin glass ground state problem is highly connected to many NP-complete and NP-hard problems, including all of Karp's 21 NP-complete problems [16, 17]. This indicates that DIRAC, which is designed for the EA spin glass ground state problem, has also the potential to solve many other NP problems.

We choose the max-cut problem as the testboard. The max-cut problem is also known as the graph partitioning problem [18], which is the canonical example of NP problems that have Ising spin glass formulations [17]. Let us consider an undirected graph $\mathcal{G} = (\mathcal{V}, \mathcal{E}, \mathcal{W})$, where $\mathcal{V}$, $\mathcal{E}$, and $\mathcal{W}$ are nodes set, edge set, edge weight set, respectively. The max-cut problem seeks to find a partition of the set $\mathcal{V}$ into two subsets such that the total weights of edges connecting the two sets are maximized.

A. Ising spin glass formulation of the max-cut problem

Here we show that the max-cut problem and the Ising spin glass ground state problem are equivalent [17]. We place an Ising spin $\sigma_i = \pm 1$ on each node $v_i \in \mathcal{V}$ in the graph, and let $+1$ and -1 denote the node belonging to either the $+$ set or the $-$ set. The max-cut problem seeks to maximize the following objective function:

$$E(W) = \frac{1}{2} \sum_{\langle i,j \rangle} (1 - \sigma_i \sigma_j) W_{ij}. \tag{7}$$

Eq. (7) can be written as:

$$E(W) = -\frac{1}{2} \sum_{\langle i,j \rangle} W_{ij} \sigma_i \sigma_j + \frac{1}{2} \sum_{\langle i,j \rangle} W_{ij} \tag{8}$$

where the second term is the total weights of all edges, which is a constant for a given graph and is denoted as C hereafter. Let $W_{ij} = -J_{ij}$, Eq. (8) can be written as:

$$E(J) = \frac{1}{2} \sum_{\langle i,j \rangle} J_{ij} \sigma_i \sigma_j + C, \tag{9}$$

Recall that finding the EA spin glass ground state is actually to minimize:

$$\mathcal{H}(J) = -\sum_{\langle i,j \rangle} J_{ij} \sigma_i \sigma_j. \tag{10}$$

It is obvious that maximizing Eq. (9) is equivalent to minimizing Eq. (10). Therefore, the max-cut problem has a clear Ising spin glass formulation.

B. Results

To apply DIRAC to solve the max-cut problem, we trained and tested DIRAC using randomly generated Barabási-Albert (BA) graphs [19] with edge weights sampled from the uniform distribution $\mathcal{U}(0, 1)$. Since there are no coordinates for general graphs, SGNN initializes degrees as nodes' input features. In particular, we set the node input feature vector $\mathbf{x}_i = (k_i, 1, 1)^\top$. The reward is defined as the cut weight change at each step. DIRAC was trained on small BA graphs and evaluated on larger instance. During the training phase, for each training iteration, DIRAC started from the empty cut-set (one side is the whole graph, the other side is an empty graph), and each step moved one node from the whole graph side to the empty graph side, until the two sides were exchanged. DIRAC^m can be designed similarly with the help of gauge transformation.

We compared DIRAC with two baseline methods: MaxcutApprox [18] and S2V-DQN [4]. The MaxcutApprox method maintains the cut set and greedily moves a node from one side to the other side of the cut if that operation results in the largest cut weight improvement. Similar as DIRAC, we also performed 1,000 runs for MaxcutApprox.

S2V-DQN is a deep learning architecture designed to solve graph combinatorial optimization problems, including the max-cut problem. It utilizes structure2vec [20] for graph representations, and Q-learning for parameters update. We adopted S2V-DQN's default setting[1] in solving the max-cut problem. Note that S2V-DQN cannot be deployed with multiple runs.

For both DIRAC and S2V-DQN, we trained them on BA graphs with 30 nodes (for one million iterations over 200,000 graphs). We then compared MaxcutApprox, the well-trained S2V-DQN and the well-trained DIRAC on larger BA graphs: 30, 50, 200, 300 and 500 nodes. For each size, we randomly generated 50 instances, and used Gurobi [21] to calculate the optimal solutions (ground truths) . We aggregated these 50 approximation ratios with respect to the best (possibly optimal) solution found by Gurobi within 1 hour as the test performance for each method. As shown in Fig. S9, DIRAC consistently outperforms the other two methods across all graph sizes, and both MaxcutApprox and DIRAC benefit a lot from multiple runs. DIRAC with multiple runs still achieves the best among all the methods, and could actually reach the ground truth on small instances (e.g., graphs with

[1] https://github.com/Hanjun-Dai/graph_comb_opt

30 and 50 nodes).

III. DIRAC'S SUPERIOR PERFORMANCES OVER OTHER METHODS UNDER DIFFERENT SCENARIOS

DIRAC is a powerful framework that could handle not only the EA spin glasses, but also a wide range of other scenarios.

A. Different topological structures

Edward-Anderson (EA) spin glasses are defined on lattice structures. We know that Belief Propagation (BP) is typically used to solve optimization problems on trees, or locally tree-like graphs. So we decided to compare DIRAC and BP on the tree-like structures. We implemented the MaxProduct algorithm [22], a variant of BP that allows us to identify the most probable configuration, i.e., the ground state in our case.

We tested MaxProduct on a cubic lattice (of side $L = 4$ and system size $N = L^3 = 64$), its spanning tree, and its spanning tree with one additional edge (termed as "loopy tree"). For each topological structure, we generated 100 instances. The results on the final energy density are shown in Fig. S12. We found that MaxProduct calculates the exact ground states (confirmed by Gurobi) on the spanning tree of the lattice, performs relatively well on the loopy tree (which is locally tree like), but performs poorly on the lattice. By contrast, DIRAC performs consistently well on the three different topological structures. We want to emphasize that here DIRAC was trained only on small lattices, but it generalizes very well to trees and loopy trees. These results suggest that DIRAC is much more versatile than BP.

B. Different hardness regimes

In the main text, we randomly generated instances of the EA spin glass model on 2D/3D/4D lattices with couplings $\{J_{ij}\}$ randomly drawn from the Gaussian distribution of zero mean and unit variance. We didn't spend additional efforts tuning the hardness of the ground state problem for the EA spin glass model. Here, to illustrate how DIRAC performs on different hardness regimes, we employ the tile planting technique (implemented

by the Chook package [23]) to generate 3D EA instances ($D = 3$, $L = 10$) with different levels of hardness, which are determined by the ratio of three different sub-lattices, denoted by F_{22}, F_{42} and F_6, respectively.

A plaquette, namely the minimal loop surrounded by four bonds, is said to be frustrated if it consists of odd number of anti-ferromagnetic bonds, because not all bonds can be satisfied at the same time. The hardness of a sub-lattice with six surfaces/plaquettes is determined by how many frustrated plaquettes it has. As shown in Fig. S13 (adopted from Fig. 2b in Ref. [23]), F_{22}, F_{42}, and F_6 have 2, 4, and 6 frustrated plaquettes, respectively, and hence correspond to increasing levels of hardness. As a result, we can adjust the hardness of the whole system by tuning the fractions of the three different sub-lattices. Generally speaking, higher fractions of F_6 sub-lattices correspond to harder instances. Note that those lattice instances with planted sub-lattices have predetermined ground state energy.

As shown in Fig. S14, we found that as the system hardness increases (from left to right), the ground state energy becomes higher, and it is indeed harder for different methods to reach the exact ground state (given the same number of initial configurations). In the "Easy" regime (Fig. S14 (a,d)), most methods can eventually reach the ground state for all instances, but DIRAC, especially DIRAC-SA, uses much fewer initial configurations than the competitive methods. For example, DIRAC-SA reaches the ground state with only 5,000 initial configurations while PT (and SA) takes 11,480 (and 20,000) initial configurations to reach the ground state, respectively. In the "Medium" regime (Fig. S14 (b,e)), only DIRAC-SA could reach the ground state (with $n_{\text{initial}} = 15,000$) , while all other methods couldn't reach it even with $n_{\text{initial}} = 20,000$. In the "Hard" regime (Fig. S14 (c,f)), although none of the presented methods could reach the ground state for all instances with up to $n_{\text{initial}} = 20,000$, DIRAC-SA reaches the lowest energy, and could obtain the ground states for some instances (Fig. S14 c). By contrast, all the other methods could not obtain the ground state for any instance we tested in this hard case. Therefore, we conclude that DIRAC (and its variants) outperforms classical methods (SA and PT) regardless of the problem hardness.

C. Different spin glass models

In main text, we applied DIRAC to EA spin glass instances which feature nearest-neighbor interactions. Here we we consider a more challenging model, i.e., the Sherrington-Kirkpatrick (SK) spin glass model [24], which features all-to-all interactions. We applied DIRAC to SK spin glass instances with couplings $\{J_{ij}\}$ randomly drawn from the Gaussian distribution of zero mean and unit variance. In particular, we trained DIRAC on small SK instances of size $N = 27$, and applied it to three larger SK instances of size $N = 64, 125, 216$. (For SK instances of such sizes, we can still use the Gurobi solver to calculate the exact ground state.) The comparison results of DIRAC (and its variants) and classical methods (SA and PT) are shown in Fig. S15. We found that DIRAC (and its variants) significantly outperforms SA and PT on the SK spin-glass model. Specifically, for those test sizes, all DIRAC variants, including DIRAC^m, DIRAC-SA and DIRAC-PT could reach the exact ground state with much fewer initial configurations than those competitive methods. For example, DIRAC-PT reaches the ground state with only 440 (Fig. S15 (a,d)) and 500 (Fig. S15 (b,e)) initial configurations on SK instances with $N = 64$ and 125, respectively. By contrast, SA and PT could not reach the ground state with even $20,000$ initial configurations. We conclude that DIRAC (and its variants) outperforms classical methods (SA and PT) on the more challenging SK spin glass model.

IV. EXISTING METHODS FOR FINDING GROUND STATES OF ISING SPIN GLASSES

In the past decades, physicists and computer scientists have developed numerous methods to calculate the ground states of Ising spin glasses. In this section, we briefly review some representative methods.

A. Exact algorithms

Finding the ground states of Ising spin glasses has been acknowledged as an NP-hard problem [17, 25–27], due to the fact that in most cases there does not exist a polynomial algorithm (which means the time complexity grows as a polynomial of the system size) to find an exact solution to the problem; namely the spin configuration with lowest energy

in the whole configuration space. That being said, we can only have exact algorithms of exponentially-increasing time complexity for general cases. It is possible to design a polynomial algorithm for some special cases, e.g., square lattice with no more than one side of periodic boundary condition or planar graphs in general.

1. Branch and Bound

The general idea of Branch and Bound is to branch the original problem into two subproblems (with a given site taking value of $+1$ or -1), and abandon those branches that can not even possibly offer the optimal solution [26]. An advantage of the Branch and Bound algorithm in the context of searching for Ising spin glass ground states is that it can find out all the states between the lowest energy and a given upper bound [26].

2. Minimum Weight Perfect Matching

For Ising spin glass on planar graphs, we have a polynomial algorithm to find the ground states, based on the observation that the max-cut problem (equivalent to the spin glass ground state problem, as discussed in Sec. II A), can be converted into the Minimum-Weight-Perfect-Matching problem [28, 29]. The latter problem is actually a standard graph optimization problem, and can be solved by existing toolboxes, e.g., Blossom V [30]. The worst time complexity of this method is $O(N^3 M)$, where N is the number of vertices and M is the number of edges.

B. Heuristic algorithms

Monte Carlo (MC)-based heuristic methods have proven to be efficient in finding the ground state of spin glasses. Of course, a trade-off between time/memory cost and the precision of the solutions (precision means how close it is to the exact solution) is always inevitable.

For a system with inverse temperature β and energy E, an attempt of perturbation to the system with energy cost ΔE will be accepted with a probability of $\min\{1, \exp(-\beta \Delta E)\}$. This is the well-known Metropolis-Hastings strategy [31], which has been leveraged in several

MC-based methods, e.g., Simulated Annealing [32], Population Annealing [33] and Parallel tempering [34–36]. For convenience, we define some terminology in the context of the Metropolis–Hastings framework. A move refers to a small perturbation of the system (which is defined to be a single spin flip in this study). A sweep refers to be N proposed random moves (per replica), where N is the total number of spins.

1. Simulated Annealing (SA)

In SA, we start from a relatively high temperature, try to do a fixed amount of MC sweeps, and then lower the system temperature step by step. We keep doing this until the system is cooled to a given temperature. The lowest energy and the corresponding spin configuration in this process are the predicted ground state energy and spin configuration, which do not necessarily coincide with that of the true ground state. We can run SA on many replicas (copies of the systems that share the same $\{J_{ij}\}$ but not necessarily the same spin configuration) to get more precise solutions.

2. Population Annealing (PA)

As the name suggests, PA performs SA on several replicas in parallel. These replicas are set to be at the same temperature and after several sweeps, there is a so-called temperature step. In the temperature step, generally we want to duplicate those replicas with low energies and eliminate those replicas with high energies, and at the same time guarantee the number of replicas does not change.

3. Parallel Tempering (PT)

This method differs from PA in the sense that the replicas are simulating at different temperatures. Similar to PA, there is also a temperature step after a fixed amount of sweeps. However, in PT, the temperature step implies the exchange between two replicas with the probability of $\min\{1, \exp[(\beta - \beta')(E - E')]\}$.

Both PA and PT are quite efficient in solving the spin glass ground state problem because they can jump out of local minimum and efficiently explore the configuration space. Another

advantage is that they are not so sensitive to the system size and dimension.

A standard MC method attempts to sample a canonical ensemble, namely infinite replicas of a system described by a specific Hamiltonian at a given temperature. Yet, limited by the computational resources, we typically can only use the Metropolis-Hastings strategy to approximate a sampling:

$$P_{\text{accept}}(\Delta E) = \min[1, \exp(-\beta \Delta E)] \tag{11}$$

Here we determine the probability of accepting a move (for example, try to flip a single spin) according to the energy change ΔE of such a move. We also call such a move the MC step. $\beta = 1/(k_B T)$ is the so-called inverse temperature, where k_B is the Boltzmann constant and usually set to be 1 for convenience, and T is the temperature describing whether the strategy prefers the move that lowers the energy.

Now we turn to a more complicated case: we sample several (but finite) replicas, fixed at different temperatures, at the same time. In such a setting, besides the move we mentioned in the context above, namely the spin flipping, we have another kind of move called the replica exchange with the acceptance probability:

$$P_{\text{accept}}(E_1, E_2; \beta_1, \beta_2) = \min\{1, \exp[(\beta_1 - \beta_2)(E_1 - E_2)]\}. \tag{12}$$

A combination of the MC steps and the replica exchanges induces the PT algorithm (also called the exchange Monte Carlo method due to the replica exchange operation [36]). There could be many possible ways to implement such an algorithm. For example, after how many MC steps do we perform a replica exchange, or, perform multiple replica exchanges? How many replicas do we need and how to set the temperatures? Considering the performance of different realizations investigated in the previous literature, we chose a specific version with the best performance, namely the "variant B" in [13], which is described in Algo. S1. Here, LISSP() refers to Local Improve based on Single-Spin fliP, which indicates the procedure that keeps flipping any spin that could lower the system energy until the energy can not be lowered any more. If the local energy minimum is smaller than the recorded lowest energy, save the minimum as the lowest energy, as well as the corresponding spin configuration.

The key idea of PT is the art of balance between low-temperature energy descent and high-temperature configuration space exploration. During each epoch, the low-temperature replica always searches for a lower-energy state. Meanwhile, the high-temperature replica

usually accepts all possible moves. At the end of each epoch, it is possible that a high-temperature replica exchanges its state with a low-temperature one, and this prevents the low-temperature replica from getting stuck in some local minimums.

C. Machine learning algorithms

Recently, several machine learning based algorithms have been developed to calculate the spin glass ground states. For example, in [37], a reinforcement-learning-controlled SA algorithm was proposed, which learns a smarter annealing schedule than the simple linear way, and facilitates the finding of the ground state. In [38], the authors leveraged the graph neural network to directly learn a mapping from the interaction matrix to the ground state configuration. The mapping is learned from small instances with known ground states to accelerate the search for the ground states of large instances. However, those algorithms could only deal with small Ising spin glass systems in three and lower dimensions, and could not even achieve accuracy close to PT. As such, we do not compare DIRAC-based methods with them in our numerical experiments.

Algorithm S1: Parallel Tempering (PT)

Input: The coupling constants $\{J_{ij}\}$ of a system with size N; N_r replicas with inverse temperatures $\beta_r = 1/[0.1 + (r-1) \times \frac{1.6-0.1}{N_\mathrm{r}-1}]$, $r = 1, 2, \ldots, N_\mathrm{r}$; and the number of epochs N_e.

Output: The lowest-energy spin configuration, as well as the lowest energy.

Randomly initialize the spin configurations of all replicas;
for epoch $= 1, 2, \ldots, N_\mathrm{e}$ **do**
    for $r = 1, 2 \ldots, N_\mathrm{r}$ **do**
        for MCstep $= 1, 2, \ldots, N$ **do**
            Randomly select a site from N sites in the replica, calculate the energy change ΔE if we flip the spin on that site;
            Accept the MCstep with the acceptance probability $P_\text{accept}(\Delta E) = \min[1, \exp(-\beta_r \Delta E)]$;
            if $\Delta E < 0$ **then**
                Compare to the lowest energy, replace it if the current one is lower;
            end
        end
    end
    Randomly select two replicas from all replicas, denoted as r_1 nad r_2;
    Accept the replica exchange with probability $P_\text{accept}(E_{r_1}, E_{r_2}; \beta_{r_1}, \beta_{r_2}) = \min\{1, \exp[(\beta_{r_1} - \beta_{r_2})(E_{r_1} - E_{r_2})]\}$;
end
LISSP();

Algorithm S2: Simulated Annealing (SA)

Input: The coupling constants $\{J_{ij}\}$ of a system with size N; the number of temperatures N_t; highest inverse temperature β_max; lowest inverse temperature β_min, the number of sweeps N_s.

Output: The lowest-energy spin configuration, as well as the lowest energy.

Randomly initialize the spin configuration;
for $t = 1, 2, \ldots, N_\mathrm{t}$ **do**
    Calculate the inverse temperature $\beta_t = (t-1) \times \frac{\beta_\mathrm{max} - \beta_\mathrm{min}}{N_\mathrm{t} - 1} + \beta_\mathrm{min}$;
    for $s = 1, 2, \ldots, N_\mathrm{s}$ **do**
        for MCstep $= 1, 2, \ldots, N$ **do**
            Randomly select a site from N sites, calculate the energy change ΔE if we flip the spin on that site;
            Accept the MCstep with the acceptance probability $P_\mathrm{accept}(\Delta E) = \min[1, \exp(-\beta_t \Delta E)]$;
            if $\Delta E < 0$ **then**
                Compare to the lowest energy, replace it if the current one is lower;
            end
        end
    end
end
LISSP();

Algorithm S3: SGNN encoder

Input: Lattice $\mathcal{G}(\mathcal{V}, \mathcal{E})$; spin's input features $\{\mathbf{x}_i \in \mathbb{R}^D, \forall v_i \in \mathcal{V}\}$, bond's input features $\{\mathbf{x}_{ij} \in \mathbb{R}^4, \forall e_{ij} \in \mathcal{E}\}$; depth K; learnable parameters $\mathbf{W}_1 \in \mathbb{R}^{d \times D}$, $\mathbf{W}_2 \in \mathbb{R}^{d \times 4}$, $\{\mathbf{W}_3, \mathbf{W}_4, \mathbf{W}_5, \mathbf{W}_6\} \in \mathbb{R}^{\frac{d}{2} \times d}$, $\mathbf{W}_7 \in \mathbb{R}^{d \times 2d}$ (D and d are the system dimension and size of embedding vectors, respectively.)

Output: Each spin's embedding vector $\mathbf{z}_i$, $\forall v_i \in \mathcal{V}$

Initialize node embedding $\mathbf{h}_i^{(0)} \leftarrow \mathrm{ReLU}(\mathbf{W}_1 \mathbf{x}_i) \in \mathbb{R}^d$, $\mathbf{h}_i^{(0)} \leftarrow \mathbf{h}_i^{(0)} / \|\mathbf{h}_i^{(0)}\|_2, \forall v_i \in \mathcal{V}$;

Initialize edge embedding $\mathbf{h}_{ij}^{(0)} \leftarrow \mathrm{ReLU}(\mathbf{W}_2 \mathbf{x}_{ij}) \in \mathbb{R}^d$, $\mathbf{h}_{ij}^{(0)} \leftarrow \mathbf{h}_{ij}^{(0)} / \|\mathbf{h}_{ij}^{(0)}\|_2, \forall e_{ij} \in \mathcal{E}$;

for $k = 1$ *to* K **do**

  for $e_{ij} \in \mathcal{E}$ **do**

    $\mathbf{h}_{ij}^{(k)} \leftarrow \mathrm{ReLU}([\mathbf{W}_3(\mathbf{h}_i^{(k-1)} + \mathbf{h}_j^{(k-1)}), \mathbf{W}_4 \mathbf{h}_{ij}^{(0)}]) \in \mathbb{R}^d$;

    $\mathbf{h}_{ij}^{(k)} \leftarrow \mathbf{h}_{ij}^{(k)} / |\mathbf{h}_{ij}^{(k)}\|_2$;

  end

  for $v_i \in \mathcal{V}$ **do**

    $\mathbf{h}_i^{(k)} \leftarrow \mathrm{ReLU}([\mathbf{W}_5 \sum_{j \in \mathcal{N}(v_i)} \mathbf{h}_{ij}^{(k)}, \mathbf{W}_6 \mathbf{h}_i^{(k-1)}]) \in \mathbb{R}^d$;

    $\mathbf{h}_i^{(k)} \leftarrow \mathbf{W}_7[\mathbf{h}_i^{(k-1)}, \mathbf{h}_i^{(k)}] \in \mathbb{R}^d$;

    $\mathbf{h}_i^{(k)} \leftarrow \mathbf{h}_i^{(k)} / \|\mathbf{h}_i^{(k)}\|_2$;

  end

end

$\mathbf{z}_i \leftarrow \mathbf{h}_i^{(K)} \in \mathbb{R}^d, \forall v_i \in V$;

Algorithm S4: Training process of DIRAC

Initialize the experience replay buffer $\mathcal{B}$ with size S_{buffer};

Parameterize the Q network with random weights $\Theta = \{\Theta_{\mathcal{E}}, \Theta_{\mathcal{D}}\}$;

Initialize the target Q network with weights $\hat{\Theta} = \Theta$;

Gen_Validation_data();

Gen_Training_data();

for episode $=$ *1 to* Ω **do**

    if episode *% C_1 == 0* **then**

        Gen_Training_data();

    end

    $\epsilon = \epsilon_{\text{final}} + \max\{0, (\epsilon_{\text{initial}} - \epsilon_{\text{final}}) \times \frac{n_\epsilon - \text{episode}}{n_\epsilon}\}$;

    PlayGame (ϵ);

    if episode *% C_2 == 0* **then**

        Test();

    end

    if episode *% C_3 == 0* **then**

        Reset $\hat{\Theta} = \Theta$;

    end

    Sample one (or mini-batch) random transition $(s_t, a_t, r_{t,t+n}, s_{t+n})$ from $\mathcal{B}$;

    Set

$$y_t = \begin{cases} r_{t,t+n}, & \text{if } s_{t+n} \text{ is the terminal state} \\ r_{t,t+n} + \max\{0, \gamma \max_{a_{t+n}} Q(s_{t+n}, a_{t+n}; \hat{\Theta})\}, & \text{otherwise} \end{cases};$$

    Perform Adam gradient descents to minimize $(y_t - Q(s_t, a_t; \Theta))^2$ to update Θ;

end

Return Θ;

Algorithm S5: PlayGame(ϵ)

Sample a random training instance from the Training Instance Pool, the number of its spins is N;

for TimeStep $t = 1$ *to* N **do**

  With probability ϵ, select a random action a_t;

  Otherwise select $a_t = \text{argmax}_a Q(s_t, a; \Theta)$ (s_t is the state at TimeStep t);

  Execute action a_t and observe reward $r(s_t, a_t, s_{t+1})$ (s_{t+1} is the next state after taking action a_t);

  if $t \geq n$ **then**

    $r_{t-n,t} = \sum_{k=0}^{n-1} \gamma^k r(s_{t-n+k}, a_{t-n+k}, s_{t-n+k+1})$;

    Store transition $(s_{t-n}, a_{t-n}, r_{t-n,t}, s_t)$ in $\mathcal{B}$;

  end

end

Algorithm S6: DIRAC-PT

Input: The coupling constants $\{J_{ij}\}$ of a system with size N; N_r replicas with inverse temperatures $\beta_r = 1/[0.1 + (r-1) \times \frac{1.6-0.1}{N_\mathrm{r}-1}]$, $r = 1, 2, \ldots, N_\mathrm{r}$; the number of epochs N_e; and two decision probabilities: p and q.

Output: The lowest-energy spin configuration, as well as the lowest energy.

Randomly initialize the spin configurations of all replicas;
for epoch $= 1, 2, \ldots, N_\mathrm{e}$ **do**
    for $r = 1, 2 \ldots, N_\mathrm{r}$ **do**
        if Rand$(0,1) \leq p$ **then**
            for MCstep $= 1, 2, \ldots, N$ **do**
                Randomly select a site from N sites in the replica, calculate the energy change ΔE if we flip the spin on that site;
                Accept the MCstep with probability $P_\mathrm{accept}(\Delta E) = \min[1, \exp(-\beta_r \Delta E)]$;
                if $\Delta E < 0$ **then**
                    Compare to the lowest energy, replace it if the current one is lower;
                end
            end
        end
        else
            Use one iteration of DIRAC[1] to lower the replica's energy;
            Calculate the energy change ΔE compared to the previous configuration;
            if $\Delta E == 0$ **then**
                Perturb each spin (N times in total) with probability $P_\mathrm{randomize} = q \times (\beta_1 - \beta_r)/(\beta_1 - \beta_{N_\mathrm{r}})$;
            end
        end
    end
    Randomly select two replicas from all replicas, denoted as r_1 and r_2;
    Accept the replica exchange with probability $P_\mathrm{accept}(E_{r_1}, E_{r_2}; \beta_{r_1}, \beta_{r_2}) = \min\{1, \exp[(\beta_{r_1} - \beta_{r_2})(E_{r_1} - E_{r_2})]\}$;
end
LISSP();

Algorithm S7: DIRAC-SA

Input: The coupling constants $\{J_{ij}\}$ of a system with size N; the number of temperatures N_t; highest inverse temperature β_max, lowest inverse temperature β_min; the number of sweeps N_s, and two decision probabilities p and q.

Output: The lowest-energy spin configuration, as well as the lowest energy.

Randomly initialize the system spin configuration;
for $t = 1, 2, \ldots, N_\mathrm{t}$ **do**
    Calculate the inverse temperature $\beta_t = (t-1) \times \frac{\beta_\mathrm{max}-\beta_\mathrm{min}}{N_\mathrm{t}-1} + \beta_\mathrm{min}$;
    for $s = 1, 2, \ldots, N_\mathrm{s}$ **do**
        if Rand$(0,1) \leq p$ **then**
            for MCstep $= 1, 2, \ldots, N$ **do**
                Randomly select a site from N sites, calculate the energy change ΔE if we flip the spin on that site;
                Accept the MCstep with the acceptance probability $P_\mathrm{accept}(\Delta E) = \min[1, \exp(-\beta_t \Delta E)]$;
                if $\Delta E < 0$ **then**
                    Compare to the lowest energy, replace it if the current one is lower;
                end
            end
        end
        else
            Use one iteration of DIRAC[1] to lower the system energy;
            Calculate the energy change ΔE compared to the previous configuration;
            if $\Delta E == 0$ **then**
                Perturb each spin (N times in total) with probability $P_\mathrm{randomize} = q \times (\beta_1 - \beta_t)/(\beta_1 - \beta_{N_\mathrm{t}})$;
            end
        end
    end
end
LISSP();

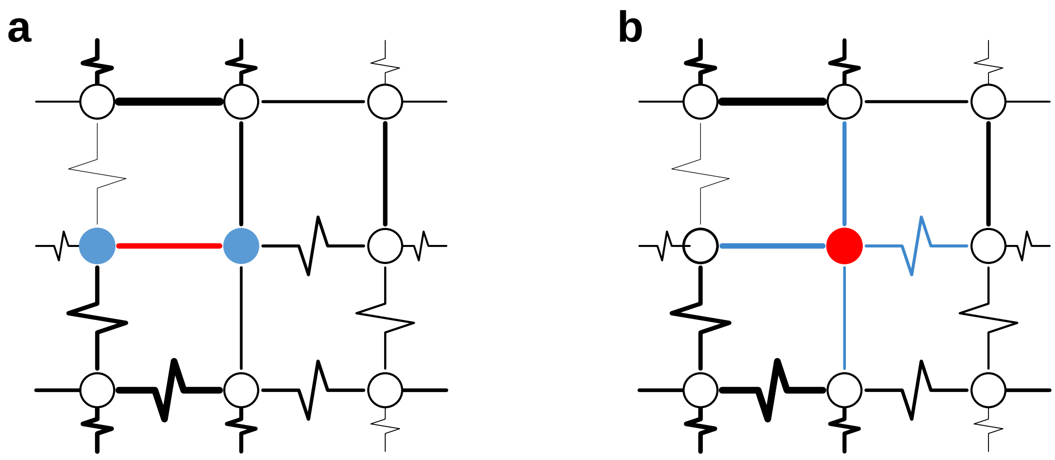

Figure S1. **Illustration of two key steps in each per-layer update (Eq. 1).** The red objects in both panels (steps) indicate the edge or node that is updated at current step, the blue objects refer to their adjacent neighbors. Eq. 1 essentially describes two key steps during each per-layer update. The first step is an edge-centric update, which updates each edge based on itself and its adjacent nodes (**a**). The second step is a node-centric update, which updates each node based on itself and its adjacent edges (**b**).

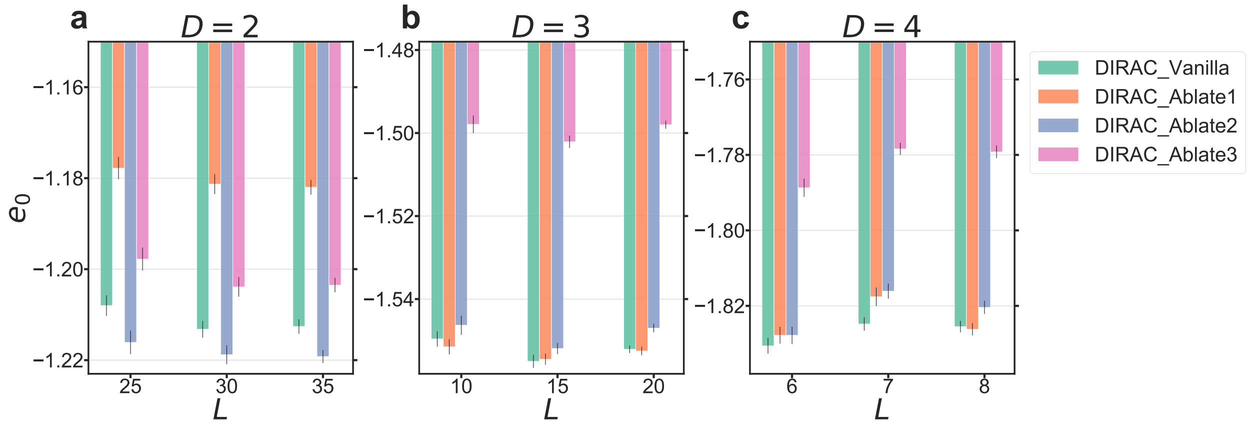

Figure S2. **Ablation study for SGNN**. We trained three ablated DIRAC agents separately and applied each agent to different sizes to explore whether all the ingredients in SGNN are really needed. DIRAC_Vanilla denotes the agent used in the present manuscript. DIRAC_Ablate1 is different in node input features, which initializes each node's feature as the constant one (the vanilla feature is node coordinates). DIRAC_Ablate2 is different in edge input features, which initializes each edge's feature as $[J_{ij}, \sigma_i, \sigma_j, 1]$ (the original feature is $[J_{ij}, \sigma_i, \sigma_i\sigma_j, 1]$). DIRAC_Ablate3 performs only the node-centric-update (by contrast DIRAC_Vanilla performs both two key updates each layer). We trained those agents on small instances, and applied them to larger ones. We evaluated the performance of each agent using the average (over 50 independent instances) "ground-state" energy density (e_0), and reported the mean (bar) and standard error of the mean (SEM) (error bar) values for each result.

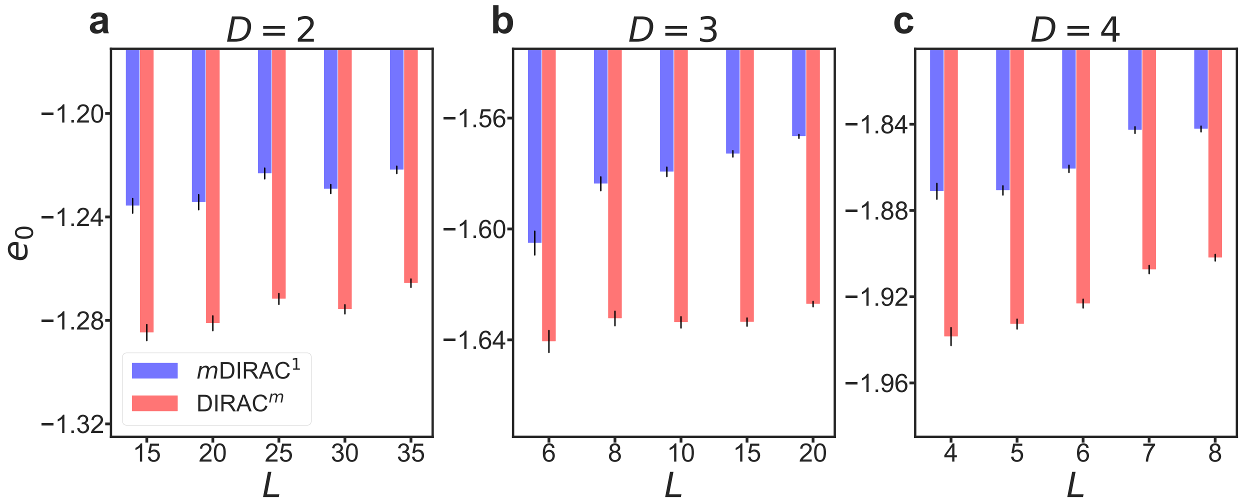

Figure S3. **DIRAC^m performs significantly better than m independent DIRAC^1.** We compared DIRAC^m and mDIRAC^1 in minimizing the energy of spin glass systems. DIRAC^m repeats m iterations of DIRAC^1, where at each iteration DIRAC^1 starts from the lowest-energy configuration from the last iteration. mDIRAC^1 also repeats m iterations of DIRAC^1, but at each iteration DIRAC^1 starts from an independent configuration. We evaluated their performances using the average (over 50 independent instances) "ground-state" energy (e_0) predicted by each method, and reported both mean (bar) and standard error of the mean (SEM) (error bar) values for each system size and method.

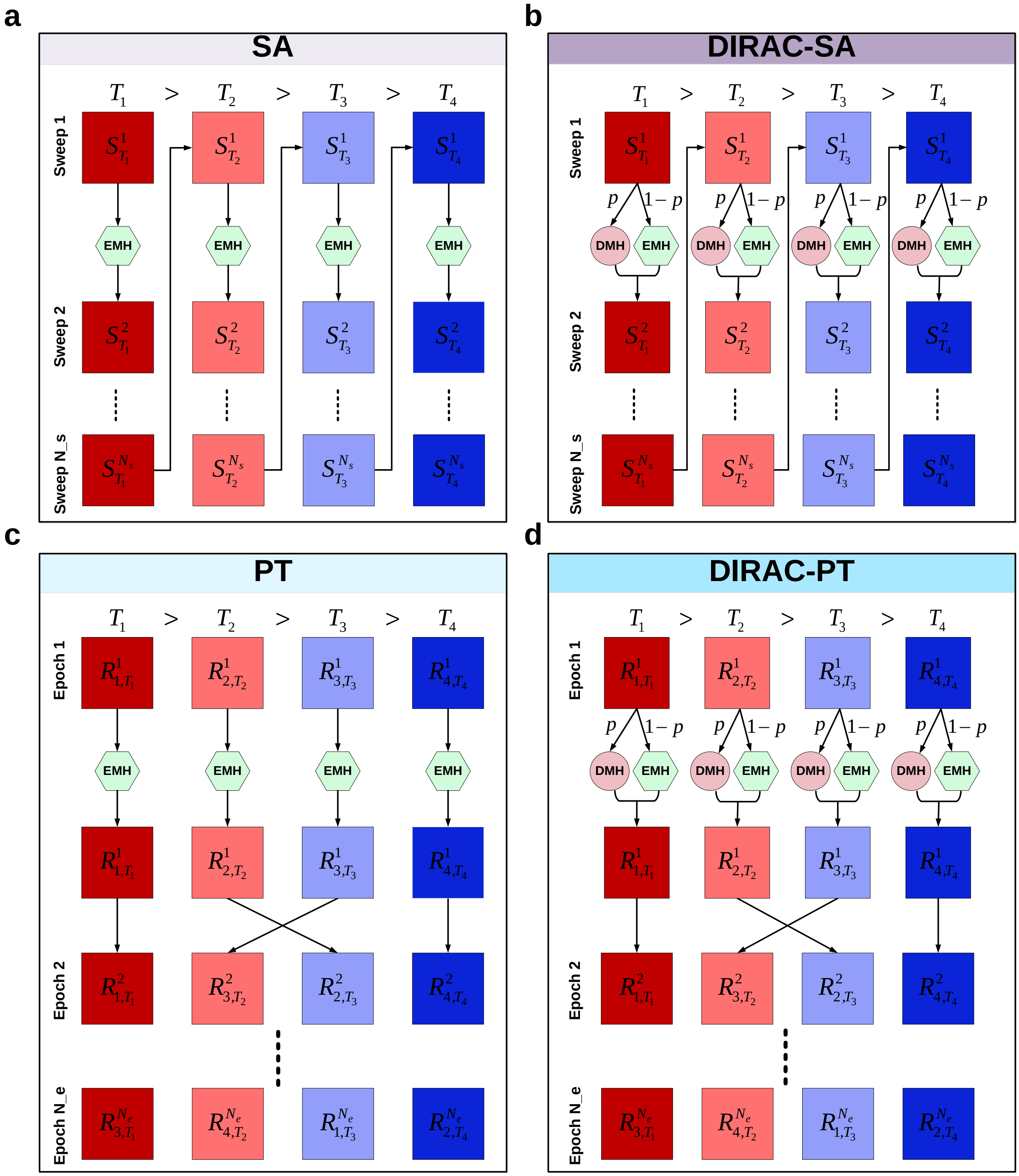

Figure S4. **Comparison of SA, PT and their DIRAC-enhanced versions: DIRAC-SA, DIRAC-PT.** Both SA and PT are annealing-based Monte-Carlo algorithms, of which the key components are both the so-called energy-based Metropolis Hastings (EMH) procedure, where a finite number of spin flips are accepted according to the Metropolis Hastings criterion. The difference between SA and PT lies in that SA runs many sweeps at different temperatures, ranging from high to low, in sequence, while PT performs a number of epochs on several replicas, which

are simulating at different temperatures, in parallel. The DIRAC-plug-in idea to enhance both algorithms is exactly the same, i.e., making the system (SA) or replicas (PT) at each iteration DIRAC-enabled. More specifically, the system (DIRAC-SA) or replicas (DIRAC-PT) at each iteration could either choose EMH or DMH to lower the system energy, the decision probability p is given empirically, here we set $p = 0.5$, indicating the two procedures can be chosen with equal chance. EMH refers to the energy-based Metropolis Hastings procedure, which uses the energy-descent operation to lower the system energy and also accept those "bad" moves (energy-up spin flips) with the Metropolis-Hastings criteria. DMH refers to the DIRAC-based Metropolis Hastings procedure, which uses one DIRAC[1] to lower the energy. If the system reaches a local energy minimum, DMH will perturb the spin configuration by flipping each spin with a temperature-dependent probability. Note that for SA and DIRAC-SA, $S^i_{T_j}$ denotes the system running sweep i at temperature T_j, one "sweep" is defined to be N random spin flips (N is the total number of spins), N_s is the number of sweeps. For PT and DIRAC-PT, R^i_{k,T_j} denotes the replica k running epoch i at temperature T_j, one "epoch" indicates the process that N_r replicas (fixed at different temperatures) perform N random flips in parallel, and in the end of each epoch, randomly select two replicas and accept the replica exchange with a certain probability (Eq. 12), N_r is the number of epochs.

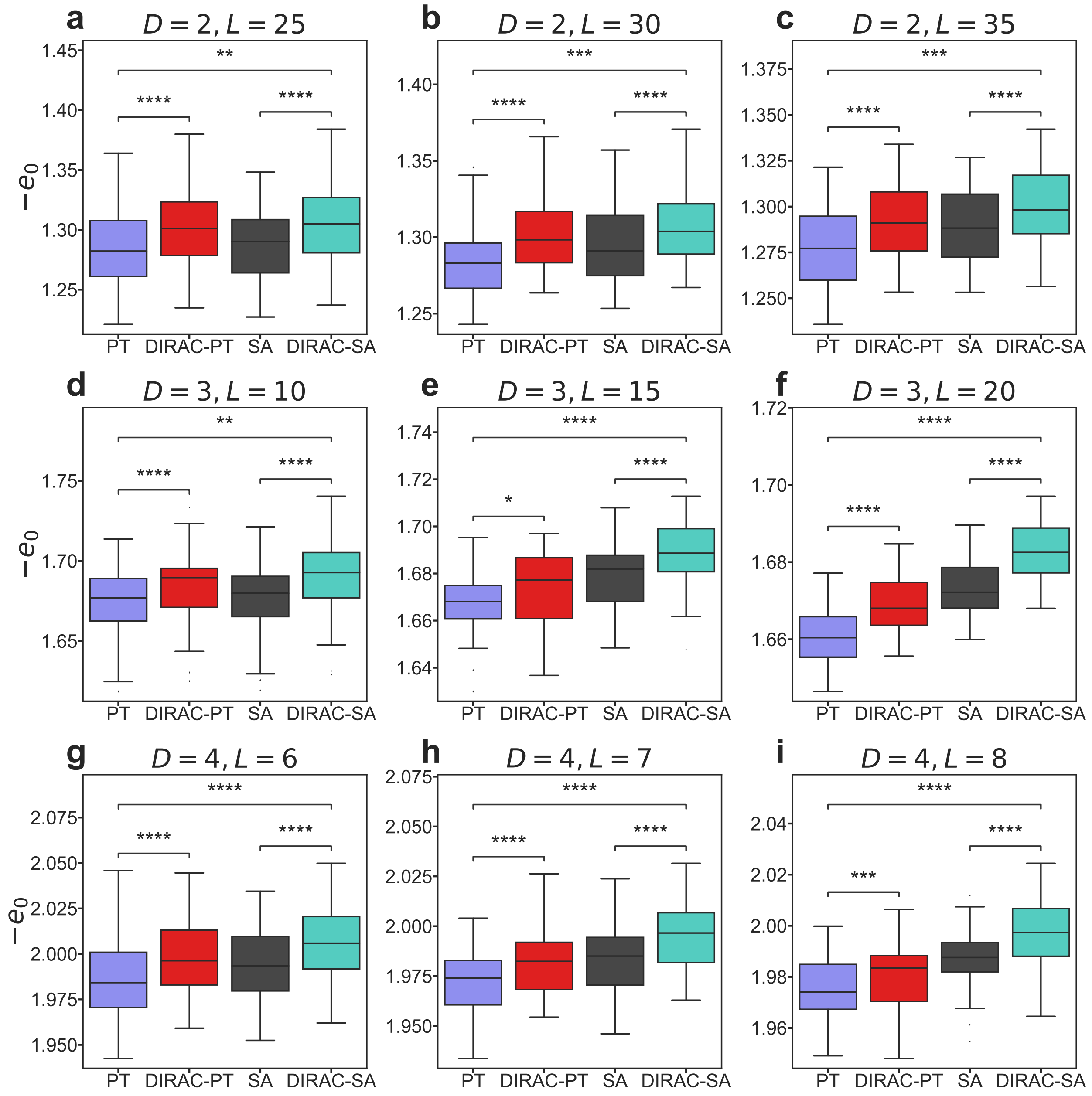

Figure S5. **Statistical test of the results in the main text Fig. 5.** We performed statistical tests between the baseline methods (SA and PT) and our DIRAC enhanced ones, so as to demonstrate our achieved gains of DIRAC-enhanced methods over those baseline methods (e.g., SA and PT) are not marginal, but statistically significant. We chose the lowest energy density results of each method (i.e., the last value of each curve in the main text Fig. 5). Here e_0 denotes the energy density. We plotted $-e_0$ in the y-axis simply for visualization purposes. For each size, we had 50 instances and reported the results in a standard box-plot, together with the p-values of the comparisons: PT vs DIRAC-PT, SA vs DIRAC-SA, PT vs. DIRAC-SA). Here, $*$ denotes $p < 0.05$, $**$ denotes $p < 0.01$, $***$ denotes $p < 0.001$, $****$ denotes $p < 0.0001$; and ns means not significant ($p >= 0.05$). Statistical test: Wilcoxon signed-ranked test.

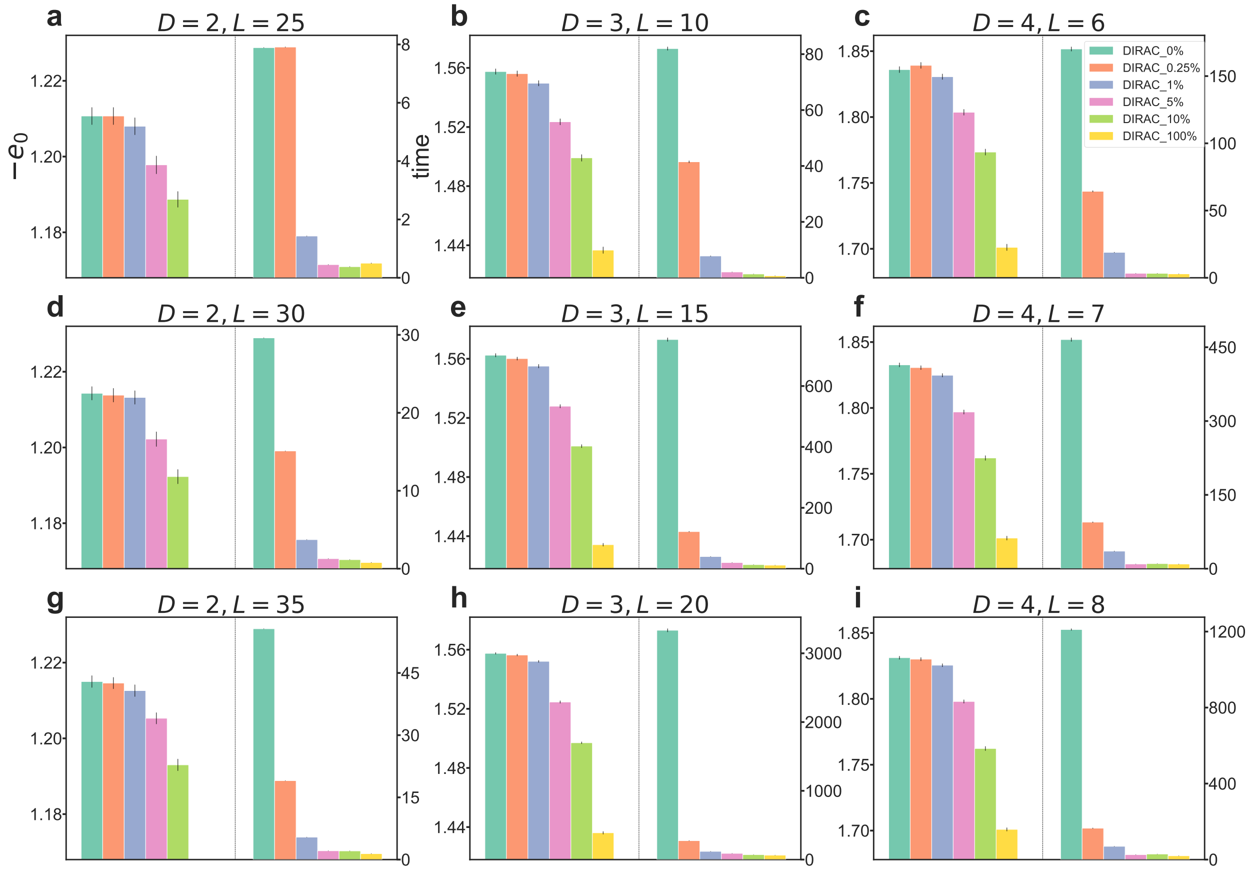

Figure S6. **Effects of batch-nodes-selection on DIRAC's performances.** During application, we found DIRAC's performances were not practically affected if we flipped a small fraction of spins with the highest Q-values at each adaptive step. We tested DIRAC (here we utilized DIRAC^1) on 2D, 3D and 4D EA spin glass instances with six different fractions, i.e., $1/N$ (flip only one spin each step), 0.25%, 1%, 5%, 10% and 100% (flip all spins at one step), and tested each case using metrics of disorder-averaged energy per spin e_0 and running time (in seconds). (**a-i**) illustrate the results on nine different system sizes, i.e., $L = 25, 30, 35$ for $D = 2$, $L = 10, 15, 20$ for $D = 3$, and $L = 6, 7, 8$ for $D = 4$. For each panel, the left panel compares the average energy (we here showed $-e_0$ for visualization purpose), and the right panel compares the running time (in seconds). Each result is averaged over 50 random instances, and the black line on top of each bar indicates the standard error of the mean (SEM). As shown here, if we flip around 1% of spins of the highest Q-values at each step, we are able to approach the one-by-one-flip performance without sacrificing too much accuracy while obtaining a significant computational speed-up.

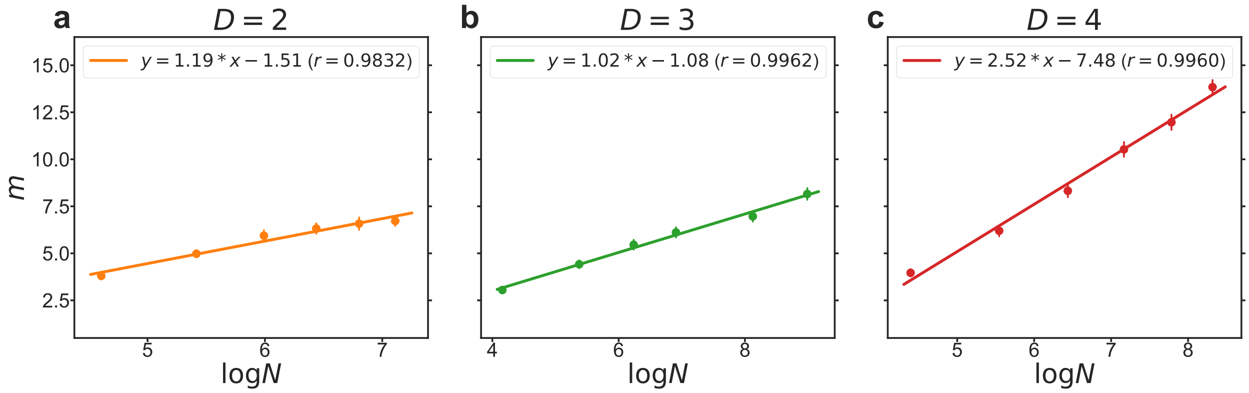

Figure S7. **Number of DIRAC^m iterations scales linearly with $\log N$.** DIRAC^m repeats m iterations of DIRAC^1. At each iteration, DIRAC^1 starts from the lowest-energy configuration from the previous iteration. Here we investigate the relationship between the number of iterations m (that is required by DIRAC^m to reach its convergence) and the logarithmic system size $\log N$. We considered 2D (**a**), 3D (**b**) and 4D (**c**) EA spin glass instances, and tested six different sizes for each dimension. Each data point was calculated by averaging over 50 random instances from the Gaussian coupling distribution $\mathcal{N}(0, 1)$. We also tried to fit a linear model to the data points, finding a high goodness of fit with correlation coefficients close to 1.

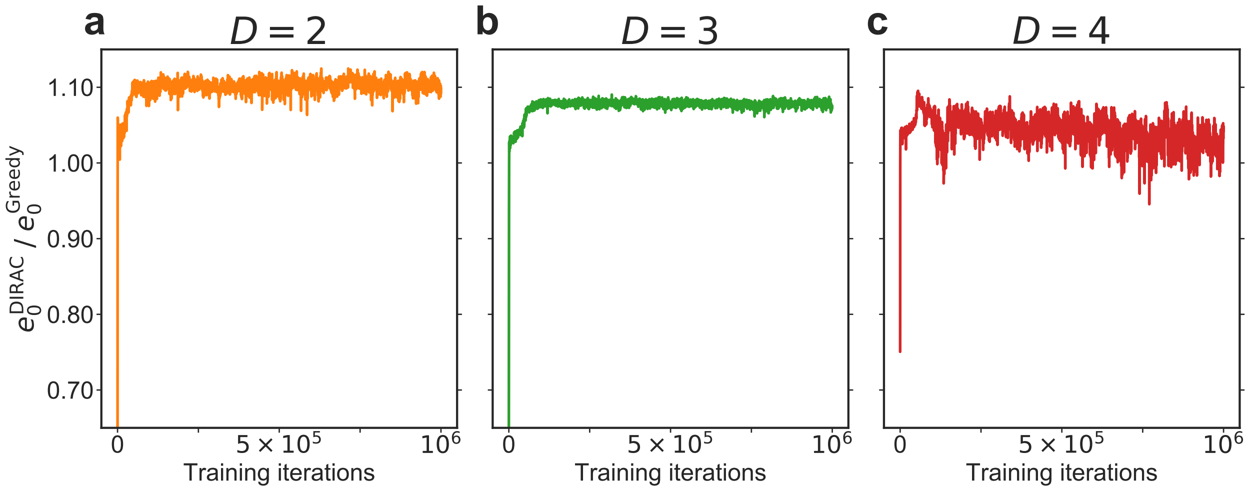

Figure S8. **DIRAC's training convergences.** We trained three DIRAC agents to calculate the ground states of 2D, 3D and 4D EA spin glass model, separately. We measured their training convergences in terms of the averaged performance over 100 validation instances. The validation performance was calculated as the approximation ratio of the predicted energy by DIRAC and the Greedy algorithm. The higher the value is, the better the model's performance. All the agents were trained using small random instances with side $L \leq 15$ for $D = 2$, $L \leq 10$ for $D = 3$ and $L \leq 6$ for $D = 4$.

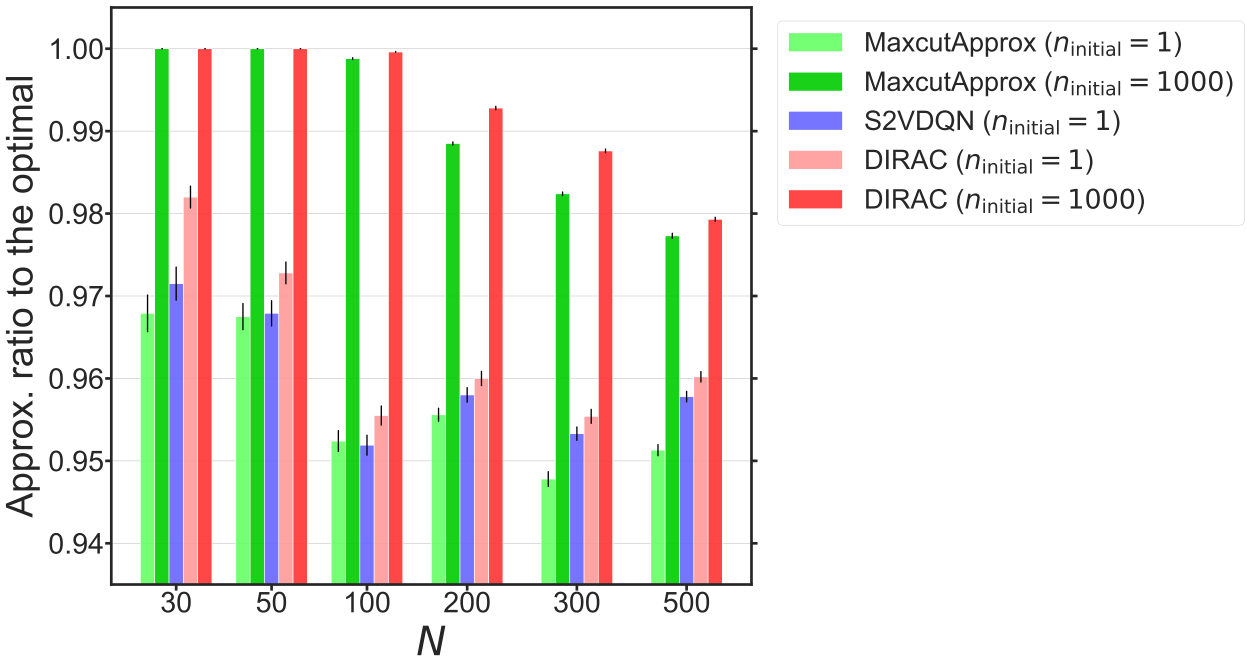

Figure S9. **DIRAC's application on the max-cut problem.** To demonstrate DIRAC's ability in solving other NP-hard problems that have Ising spin glass formulations, we focused on the max-cut problem, a well-known NP-hard problem that can be mapped to the spin glass ground state problem [17]. Under the DIRAC framework, we trained and tested the RL agent using randomly generated Barabási-Albert (BA) graphs [19] with edge weights sampled from the uniform distribution $\mathcal{U}(0, 1)$. We compared DIRAC with MaxcutApprox [18] (which maintains the cut set and moves a node from one side to the other side of the cut if that operation results in the cut weight improvement) and S2V-DQN [4] (which is another RL framework that is designed to solve the max-cut problem). Both S2V-DQN and DIRAC were trained on BA graphs with 30 nodes, and then evaluated on larger scales: 30, 50, 100, 200, 300 and 500 nodes. For each scale, we randomly generated 50 instances, and used the exact solver Gurobi [21] to get their optimal solutions. All reported approximation ratios were with respect to the best (possibly optimal) solution found by Gurobi within 1 hour. Since both MaxcutApprox and DIRAC could start from any random cut set, whilst S2V-DQN could only start from an empty cut set. We started from multiple initial states (set 1000 here) for MaxcutApprox and DIRAC, and chose the best one among all. We found that DIRAC outperforms the other two methods across all test scales, and both MaxcutApprox and DIRAC benefit a lot from multiple initial states. DIRAC with multiple initial states consistently achieves the best results among all others, and could reach the ground truth on small instances (e.g., graphs with 30 and 50 nodes).

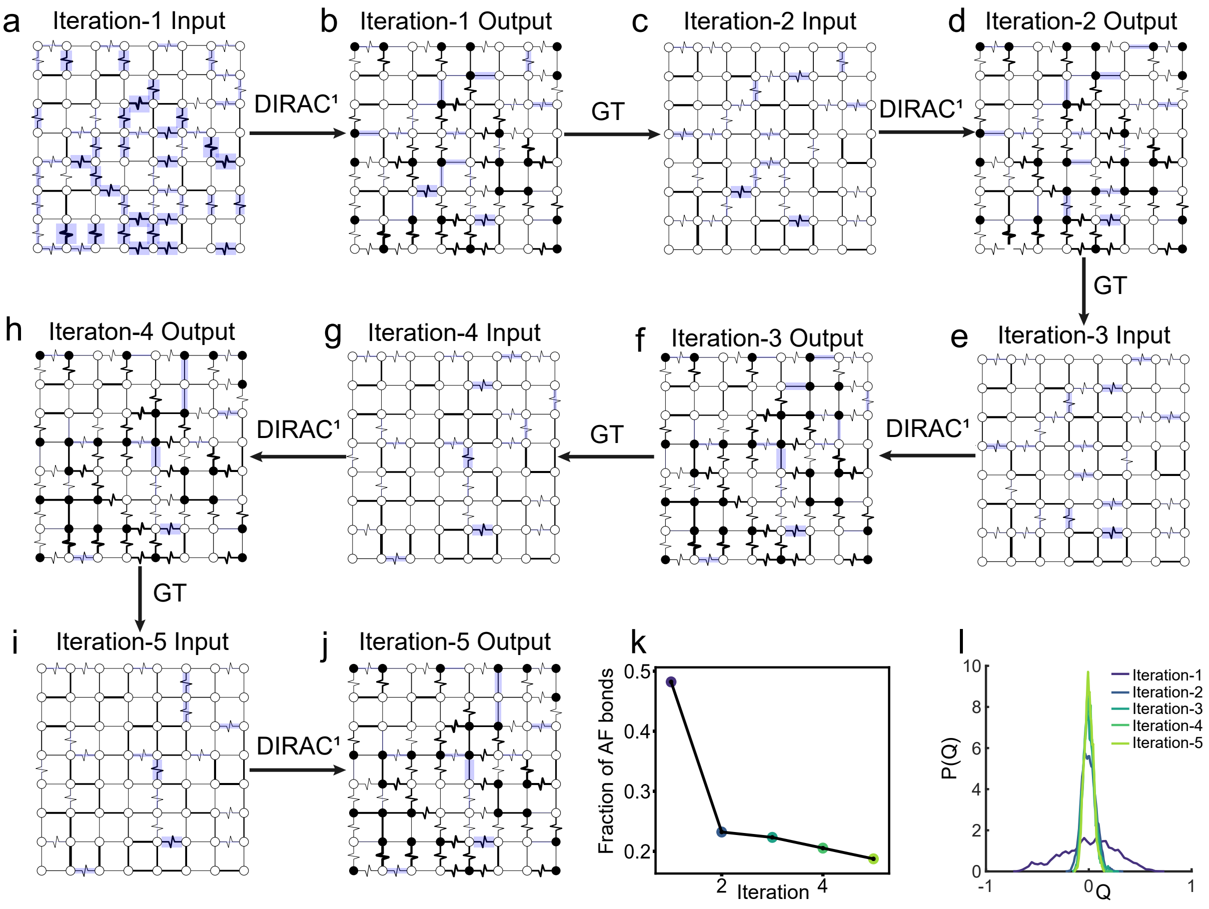

Figure S10. **An illustration of the workflow of DIRAC^m.** We applied DIRAC^m on a random 8×8 EA spin glass instance, and showed the instance state at each iteration. For each iteration, we applied DIRAC^1 to lower the energy, then we performed gauge transformation (GT) so that the configuration went back to the all-spins-up configuration while the energy remained the same. We noticed that the number of anti-ferromagnetic bonds (more precisely, the absolute sum of all negative J_{ij} values) decreased in the end of each iteration. We repeated the process until the energy saturated. In this case, DIRAC^m completed the calculation within five iterations (**a-j**), i.e., $m = 5$. Note that the spin configuration shown in (**h**) and (**j**) are exactly the same, which are both the exact ground state configuration of this instance. We found that: (1) the fraction of anti-ferromagnetic bonds in the gauge transformed instances kept decreasing (**k**); (2) the Q-value distribution became more homogeneous (**l**). Note that here the Q-values were centered (with mean value equals to zero) for comparison purpose.

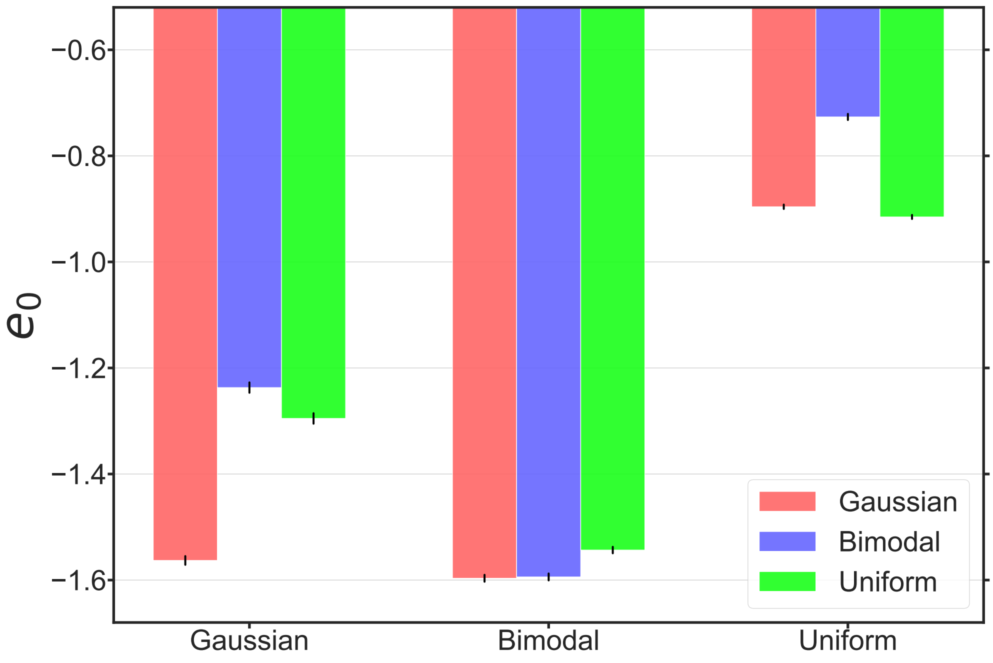

Figure S11. **DIRAC's performances on different testing coupling distributions using different training coupling distributions.** In this work, we typically trained and tested DIRAC using EA spin glass instances with couplings $\{J_{ij}\}$ sampled from the Gaussian distribution of zero mean and unit variance. Here we tested two other distributions, including discrete Bimodal distribution (coupling strength is either +1 or -1) and continuous Uniform distribution (coupling strength is a continuous value sampled from $\mathcal{U}(-1, 1)$). We trained DIRAC using different coupling distributions and evaluate its performance (using the DIRAC^1 strategy) on three test coupling distributions. Both training and test instances were randomly generated with the EA spin glass model ($D = 3$, $L = 4$), and each result was averaged over 50 test instances, error bars indicate the standard error of the mean (SEM). We found that training DIRAC using the same coupling distribution as the test instances often yields the best performance, and DIRAC trained with the Gaussian coupling distribution generalizes better than trained with the other two coupling distributions.

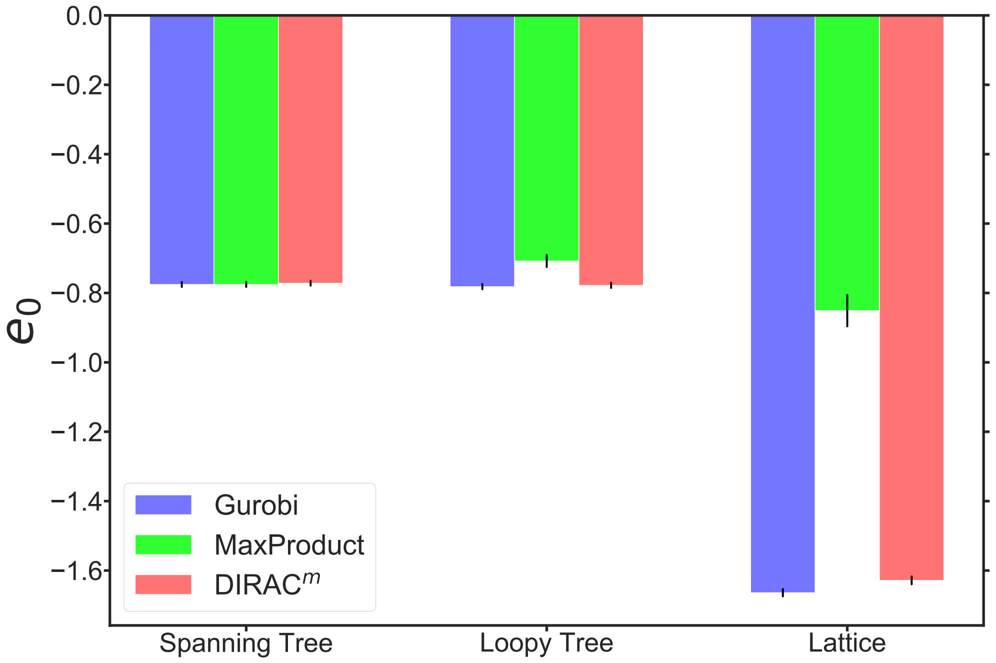

Figure S12. **Comparing the performance of DIRAC and Belief Propagation in solving the ground state problem of the spin glasses on different topological structures.** The MaxProduct algorithm[39] is a variant of Belief Propagation (BP) algorithm. We compared DIRAC (here we used the DIRAC^m strategy) and MaxProduct on the cubic lattice ($D = 3, L = 4$), its spanning tree, and its spanning tree with one additional edge (termed as the loopy tree). The energy density e_0 (averaged over 100 independent instances) is shown here. For the MaxProduct algorithm, the inverse temperature β is a hyper-parameter. Here we set $\beta = 10$, which seems to be better than other values we tried ($\beta = 0.1, 1, 5$).

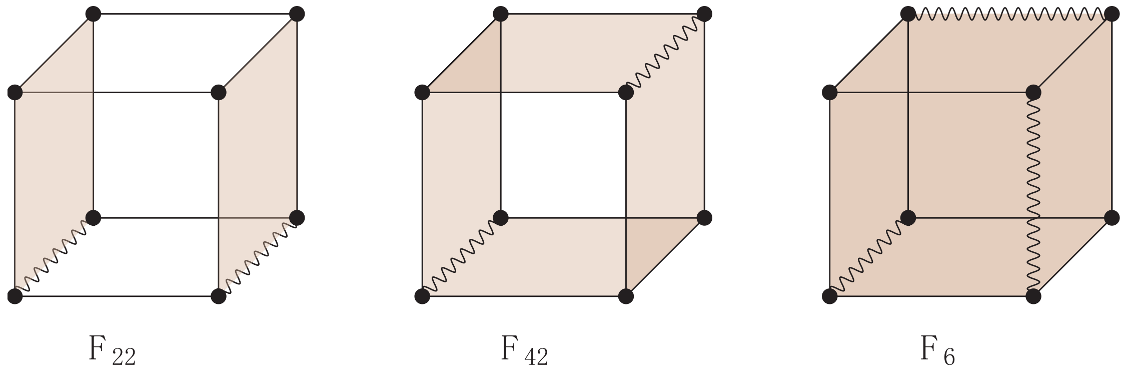

Figure S13. **Three types of sub-lattices to "plant" a spin glass instance with desired hardness.** We show three types of sub-lattices, with increasing "frustration" from left to right. Each sub-lattice has six surfaces, or plaquettes. A plaquette is said to be frustrated, if it contains odd number of anti-ferromagnetic bonds, in which case not all bonds can be satisfied at the same time. F_{22}, F_{42} and F_6 have $2, 4, 6$ frustrated plaquettes respectively. Using the planting technique introduced in Ref. [23], we can generate an instance with desired hardness, by tuning the fractions of the three sub-lattices.

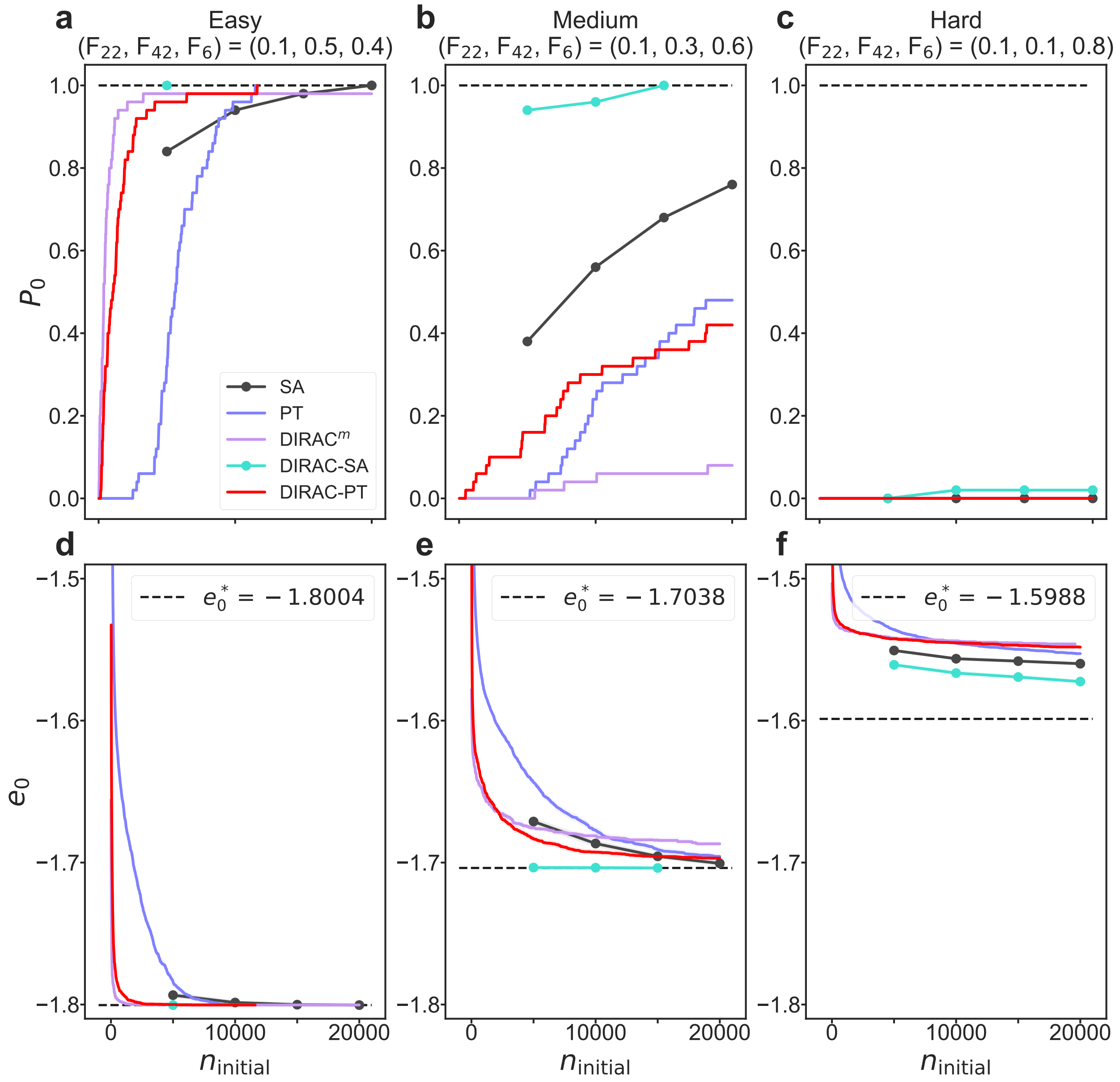

Figure S14. **Comparing the performance of DIRAC-based methods and competitive methods in solving the spin glass ground state problem with different levels of hardness.** Those spin glass instances ($D = 3$, $L = 10$) were obtained by the tile planting technique implemented by the Chook package[23]. The hardness was tuned by varying the fractions of the three different sub-lattices. Here we fixed the fraction of F_{22} and increased the fraction of F_6 to increase the hardness from Easy (**a, d**), Medium (**b, e**), to Hard (**c, f**). We compared the P_0 curve and e_0 curve of DIRAC-based methods and competitive methods on these instances with different levels of hardness. We plotted both P_0 and e_0 as a function of the number of different initial configurations (n_{initial}). For each type of instances, we ran at most $n_{\text{initial}} = 20,000$ initial configurations. At a

given n_{initial}, P_0 was calculated as the fraction of 50 random instances for which the ground state was found (and confirmed by Gurobi), and e_0 was calculated as the average lowest energy (among all runs with n_{initial} initial configurations) over the same 50 instances. For e_0 curve, we showed the results of mean and standard error of the mean (SEM) (shaded area, comparable with the line width or data point symbol size in this figure). The black dashed lines are the ground truth energy computed simultaneously when the instances were generated by the Chook package. Since in our implementations, each result of SA and DIRAC-SA was calculated using 5,000 initial configurations and we stopped the calculation when P_0 reaches 1, their curves actually consist of very few (up to 4) scatter points.

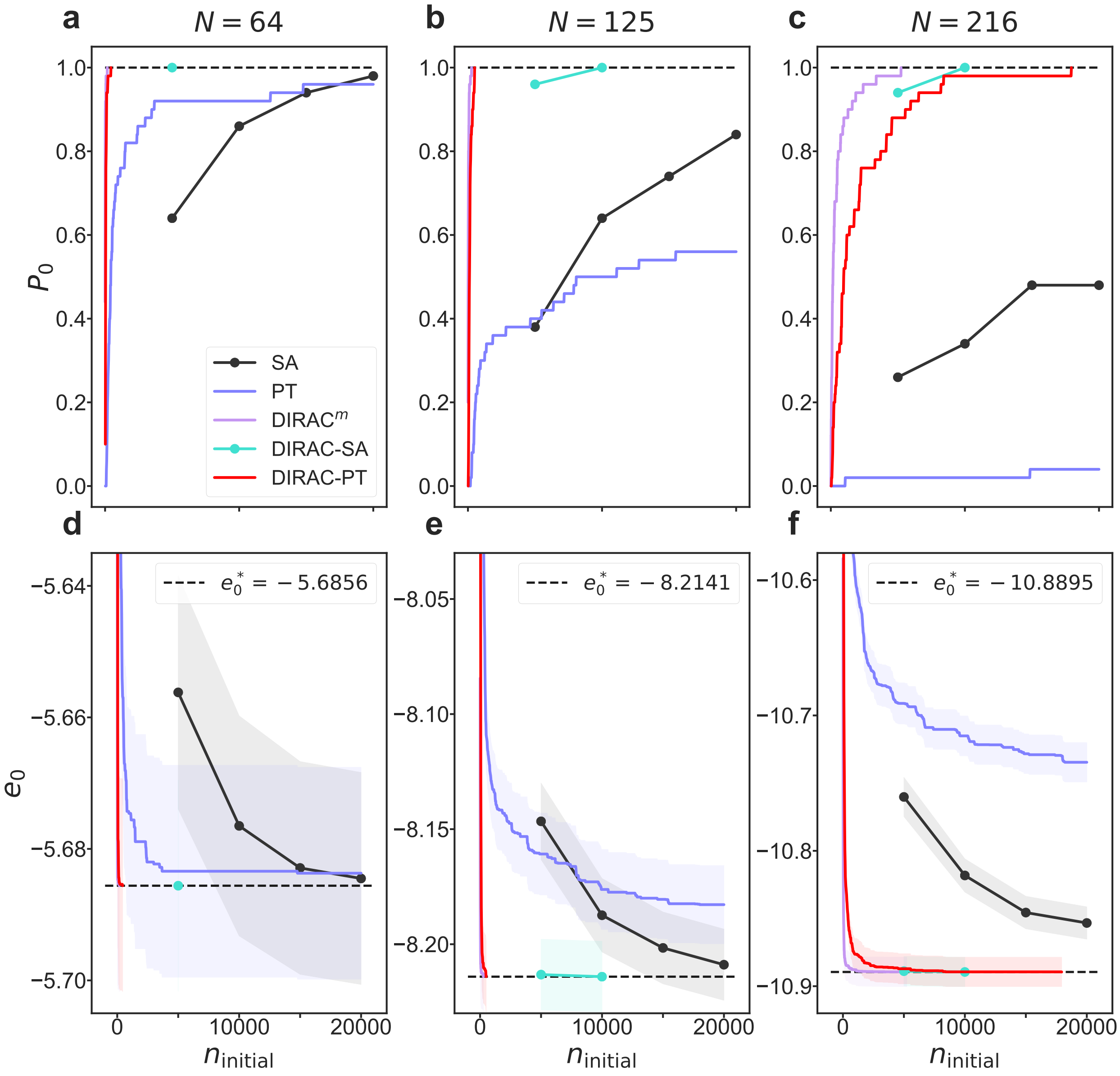

Figure S15. **Comparing the performance of DIRAC-based methods and competitive methods in solving the ground state problem of the Sherrington-Kirkpatrick (SK) spin-glass model.** We compared the P_0 curve and e_0 curve of DIRAC-based methods and competitive methods on SK instances of size $N = 64$ (**a, d**), 125 (**b, e**) and 216 (**c, f**). P_0 is the probability of finding the ground state, and e_0 is the disorder averaged "ground-state"energy per spin predicted by a method. We plotted both P_0 and e_0 as a function of the number of different initial configurations (n_{initial}). For each system size, we ran at most $n_{\text{initial}} = 20,000$ initial configurations. At a given n_{initial}, P_0 was calculated as the fraction of 50 random instances for which the ground state is found (and confirmed by Gurobi), and e_0 was calculated as the average

lowest energy (among all runs with n_{initial} initial configurations) over the same 50 instances. For e_0 curve, we showed the results of mean and standard error of the mean (SEM) (shaded area). The black dashed lines denote the exact ground state energy (averaged over the 50 instances) calculated by Gurobi. Since in our implementations, each result of SA and DIRAC-SA was calculated using 5,000 initial configurations and we stopped the calculation when P_0 reaches 1, their curves actually consist of very few scatter points.

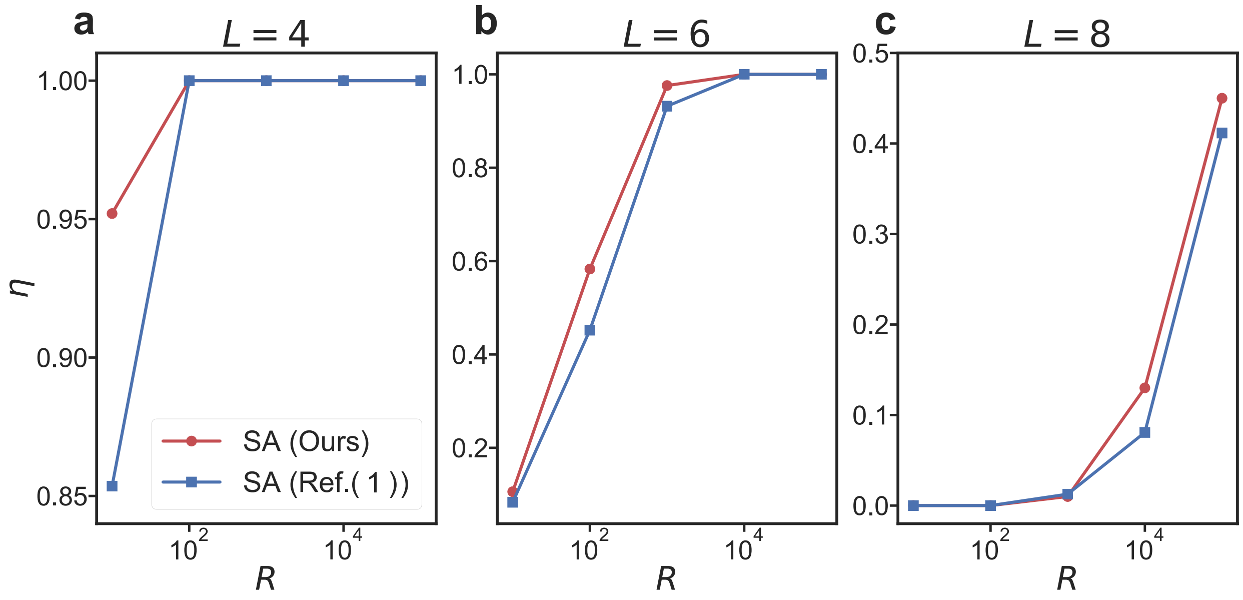

Figure S16. **Our SA implementation can reproduce results reported in the literature.** Here we compared the performance of our SA implementation and what was reported in Ref. [25] on 3D EA spin glass instances with $L = 4, 6, 8$. The results for $L = 10$ were not shown here because this size is too large for the exact solver Gurobi to calculate the ground state within tolerable computing time. We emphasize that even though the result for $L = 10$ was shown in Ref. [25], the authors actually did not directly use any exact solver to confirm all the "ground states" they found. Using the same symbols as in Ref. [25], here the horizontal axis is the number of independent runs, denoted as R. In both our and Ref. [25]'s SA implementation, one independent run consists of 5,000 initial configurations. The vertical axis is the ratio of finding the ground state in multiple instances, denoted as η (same as P_0 in our notation). All the ground states are confirmed by the exact solver Gurobi. Blue and red dots represent the results of Ref. [25] and ours respectively. Using the identical parameter setting as in Fig. 6 from Ref. [25], we set each independent run to include 101 different temperatures and 10 sweeps at each temperature, namely $N_\mathrm{t} = 101, N_s = 10$. The number of instances we calculated are $1000, 1000, 100$ for $L = 4, 6, 8$, respectively.

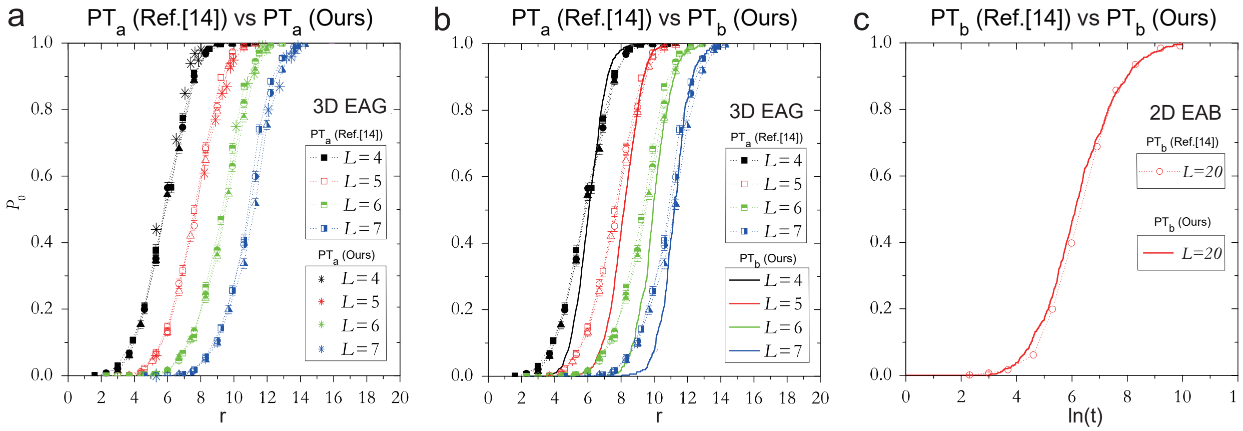

Figure S17. **Our PT implementations can reproduce results reported in the literature.** **a**, The dot lines with squares, triangles and circles show the P_0 curves obtained by the variant A of Parallel Tempering (denoted as PT$_a$) reported in Ref. [13], with different number of replicas (m) and different number of initial configurations (t, equivalent to $n_{\text{initial}}/2.3$ for variant B and $n_{\text{initial}}/2$ for variant A in our notation, where the factors of 2.3 and 2 came from Ref. [13]). The star dots represent the results of our PT$_a$ implementation with the number of replicas $m = 20$. The x-axis represents $r \equiv \ln(mt)$. **b**, We directly compared the P_0 curve of our PT$_b$ implementation with that of PT$_b$ reported in Ref. [13], which is for 2D EA spin glasses with binary couplings (i.e., J_{ij} randomly chosen from $\{+1, -1\}$) and $L = 20$, and the number of replicas $m = 20$. Note that in this case, the horizontal axis is $\ln(t)$, rather than $r \equiv \ln(mt)$. Unfortunately, Ref. [13] did not demonstrate any figures on the P_0 results of PT$_b$ from 3D EA spin glass instances with Gaussian couplings, so we cannot directly compare our PT$_b$ implementation with theirs in this circumstance.

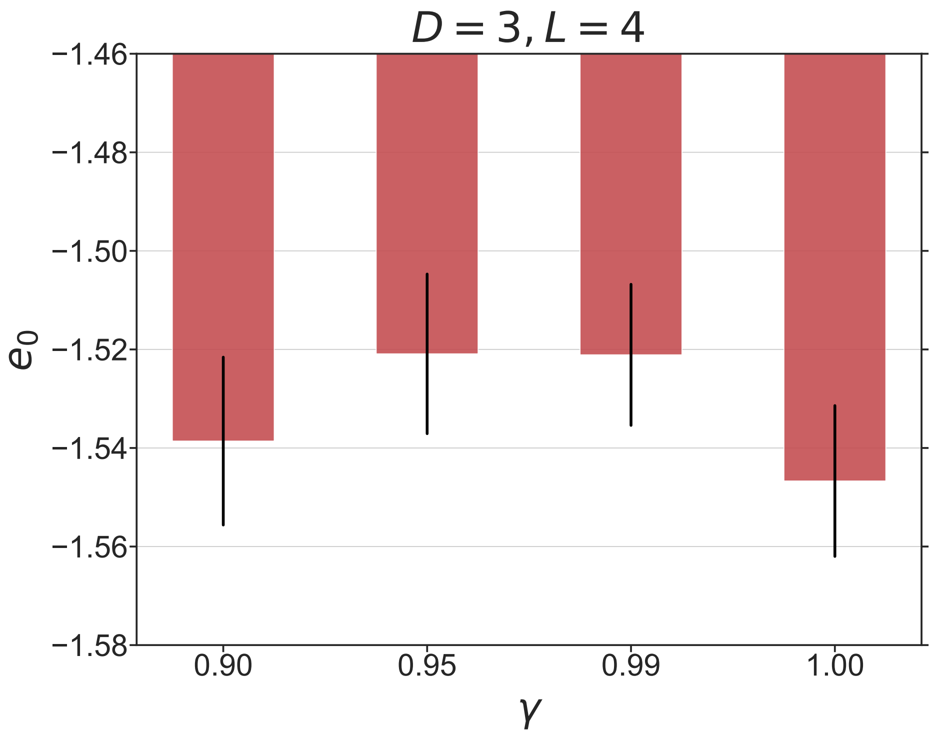

Figure S18. **Impact of the discount factor γ on DIRAC's performance.** We trained different DIRAC agents (on cubic lattices of side $L = 4$) using different discount factor values ($\gamma = 0.9, 0.95, 0.99, 1.0$) and then applied those trained agents (using the DIRAC[1] strategy) on the test instances with the same training size ($D = 3, L = 4$). e_0 denotes the disordered averaged (over 50 independent instances) "ground-state" energy per spin (predicted by DIRAC[1]). We reported the mean (bars) and standard error of the mean (SEM) (error bars) values of e_0.

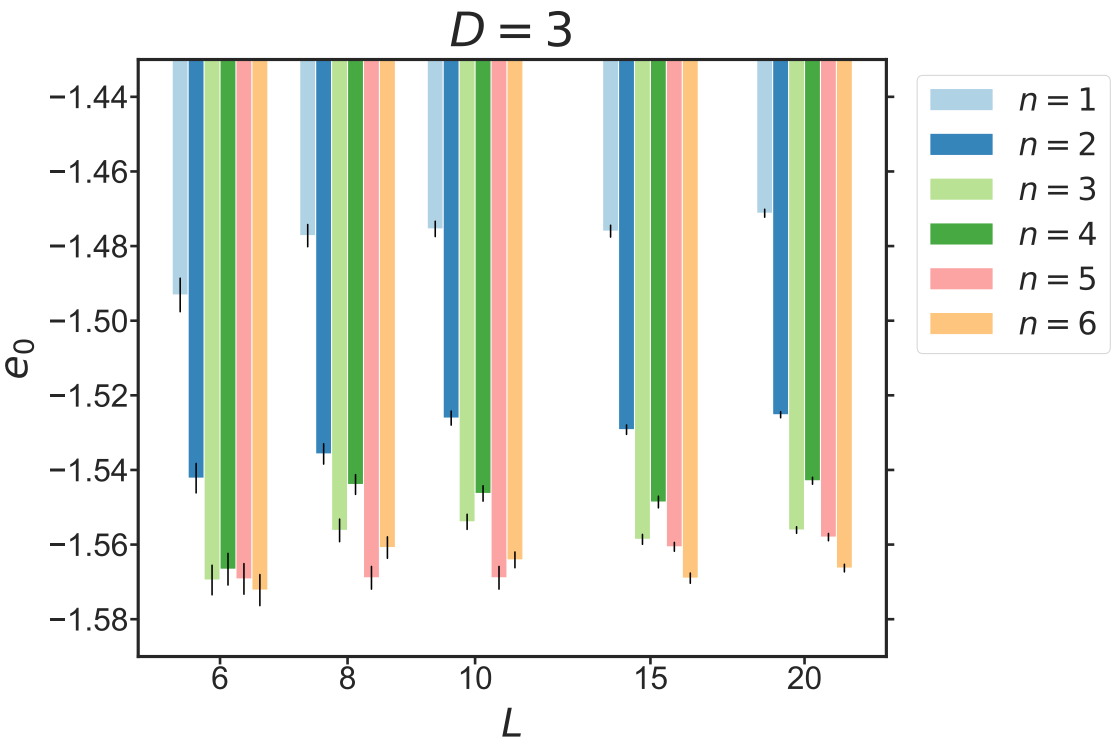

Figure S19. **Impact of the delay reward parameter n on DIRAC's performance.** We trained different DIRAC agents (on cubic lattices of side $L = 4$) using different delay reward parameter ($n = 1, 2, 3, 4, 5, 6$) and then applied (using the DIRAC[1] strategy) those trained agents on larger instances of different sides ($L = 6, 8, 10, 15, 20$). e_0 denotes the disordered averaged (over 50 independent instances) "ground-state" energy per spin (predicted by DIRAC[1]). We reported the mean (bars) and standard error of the mean (SEM) (error bars) values of e_0.

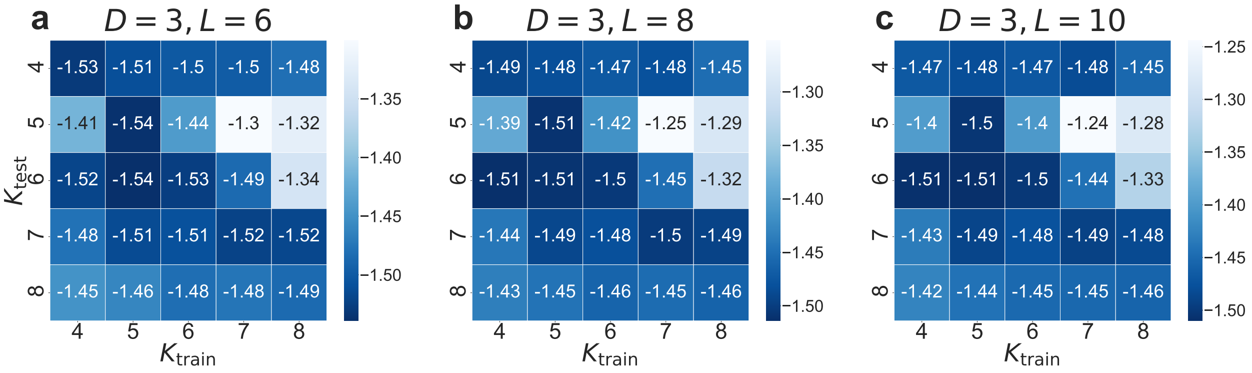

Figure S20. **Training DIRAC using the same number of message passing steps as that in the application instances yields the best performance.** We trained five DIRAC[1] agents (on the 3D systems) with different number of message passing steps ($K = 4, 5, 6, 7, 8$), and then applied each agent to different sizes ($L = 6, 8, 10$ for $D = 3$) with varying K values ($K = 4, 5, 6, 7, 8$). Since our SGNN is designed to share the same set of parameters across different layers, the trained agent could be applied using different K values. We evaluated their performances using the average (over 50 independent instances) "ground-state" energy per spin (e_0) predicted by each model, and reported the values of mean in the heatmaps.

Table S1. **Time complexity analysis.**

Method	Time complexity	Description
Greedy	$O(N)$	N is the number of spins;
SA, DIRAC-SA	$O(NN_{\mathrm{s}}N_{\mathrm{t}})$	N is the number of spins, N_{s} is the number of sweeps, N_{t} is the number of temperatures;
PT, DIRAC-PT	$O(NN_{\mathrm{e}}N_{\mathrm{r}})$	N is the number of spins, N_{e} is the number of epochs, N_{r} is the number of temperatures(replicas);
DIRAC^1	$O(N)$	N is the number of spins;
DIRAC^m	$O(mN)$	N is number of spins, m is the number of DIRAC^1 iterations, which scales linearly with $\log N$ (based on numerical calculations).

Table S2: **List of hyper-parameters, their values and brief descriptions.** Here, the first 13 hyper-parameters were used in basic DIRAC methods (i.e., DIRAC^1, DIRAC^m). Those hyper-parameter values were selected based on preliminary results on small instances and then fixed for large ones. We did not perform a systematic grid search to obtain the optimal set of hyper-parameter values. Hence, the performance of all DIRAC-based methods presented in this work is very conservative. For competitive algorithms SA and PT, their hyper-parameter values have been optimized in Ref. [13] and Ref. [25], and hence we just adopted those values in our SA and PT implementations, as well as the respective DIRAC-enhanced versions (DIRAC-SA and DIRAC-PT).

**Hyper-parameter**	**Value**	**Description**
S_{buffer}	5×10^4	memory size of the experience replay buffer $\mathcal{B}$
α	10^{-4}	learning rate used by the Adam optimizer
d	64	size of the node embedding vector
K	5	number of message-passing steps in the encoder SGNN
Ω	10^6	maximum episodes for the training process
γ	1	discount factor used in Q-learning update
n	5	number of reward delay steps for Q-learning algorithm
$\epsilon_{\text{initial}}$	1	initial value of ϵ in ϵ-greedy exploration
ϵ_{final}	5×10^{-2}	final value of ϵ in ϵ-greedy exploration
n_ϵ	5×10^4	number of steps over which the initial value of ϵ is linearly annealed to its final value

Continued on next page

Table S2 – continued from previous page

Hyper-parameter	Value	Description
C_1	5×10^3	number of episodes after which to generate training samples
C_2	300	number of episodes after which to test the trained model at current stage
C_3	10^3	number of episodes after which to copy the Q parameters to the target Q-network
N_r	20	number of replicas in PT and DIRAC-PT
p	0.5	probability of deciding EMH or DMH in DIRAC-SA and DIRAC-PT
q	0.5	magnitude of controlling the spin configuration perturbation in DMH
N_t	100	number of temperatures in SA and DIRAC-SA
$\beta_\mathrm{max}, \beta_\mathrm{min}$	5.0, 0.0	highest and lowest inverse temperatures in SA and DIRAC-SA
N_s	50	number of sweeps in SA and DIRAC-SA

SUPPLEMENTARY REFERENCES

[1] Kipf, T. N. & Welling, M. Semi-supervised classification with graph convolutional networks. In *International Conference on Learning Representations* (2017).

[2] Khalil, E., Dai, H., Zhang, Y., Dilkina, B. & Song, L. Learning combinatorial optimization algorithms over graphs. In *Advances in Neural Information Processing Systems*, 6348–6358 (2017).

[3] Hamilton, W., Ying, Z. & Leskovec, J. Inductive representation learning on large graphs. In *Advances in Neural Information Processing Systems*, 1024–1034 (2017).

[4] Khalil, E., Dai, H., Zhang, Y., Dilkina, B. & Song, L. Learning combinatorial optimization algorithms over graphs. In *Advances in Neural Information Processing Systems*, 6348–6358 (2017).

[5] Velickovic, P. *et al.* Graph attention networks. In *International Conference on Learning Representations* (2018).

[6] Fan, C., Zeng, L., Sun, Y. & Liu, Y.-Y. Finding key players in complex networks through deep reinforcement learning. *Nature Machine Intelligence* **2**, 317–324 (2020).

[7] Ying, Z. *et al.* Hierarchical graph representation learning with differentiable pooling. In *Advances in Neural Information Processing Systems*, 4800–4810 (2018).

[8] Mnih, V. *et al.* Human-level control through deep reinforcement learning. *Nature* **518**, 529–533 (2015).

[9] Sutton, R. S., McAllester, D. A., Singh, S. P. & Mansour, Y. Policy gradient methods for reinforcement learning with function approximation. In *Advances in Neural Information Processing Systems*, 1057–1063 (2000).

[10] Sutton, R. S. & Barto, A. G. *Reinforcement learning: An introduction* (MIT press, 2018).

[11] Kingma, D. P. & Ba, J. Adam: A method for stochastic optimization. *arXiv preprint arXiv:1412.6980* (2014).

[12] Wegner, F. J. Duality in generalized ising models and phase transitions without local order parameter. *Journal of Mathematical Physics* **12**, 2259 (1971).

[13] Romá, F., Risau-Gusman, S., Ramirez-Pastor, A. J., Nieto, F. & Vogel, E. E. The ground

state energy of the edwards–anderson spin glass model with a parallel tempering monte carlo algorithm. *Physica A: Statistical Mechanics and its Applications* **388**, 2821–2838 (2009).

[14] Gilmer, J., Schoenholz, S. S., Riley, P. F., Vinyals, O. & Dahl, G. E. Neural message passing for quantum chemistry. In *International Conference on Machine Learning*, 1263–1272 (PMLR, 2017).

[15] Xu, K., Hu, W., Leskovec, J. & Jegelka, S. How powerful are graph neural networks? In *International Conference on Learning Representations* (2018).

[16] Karp, R. M. Reducibility among combinatorial problems. In *Complexity of Computer Computations*, 85–103 (Springer, 1972).

[17] Lucas, A. Ising formulations of many np problems. *Frontiers in Physics* **2**, 5 (2014).

[18] Kleinberg, J. & Tardos, E. *Algorithm design* (Pearson Education India, 2006).

[19] Barabási, A.-L. & Albert, R. Emergence of scaling in random networks. *Science* **286**, 509–512 (1999).

[20] Dai, H., Dai, B. & Song, L. Discriminative embeddings of latent variable models for structured data. In *International Conference on Machine Learning*, 2702–2711 (2016).

[21] Gurobi Optimization, L. Gurobi optimizer reference manual (2021).

[22] Mezard, M. & Montanari, A. *Information, physics, and computation* (Oxford University Press, 2009).

[23] Perera, D. *et al.* Chook–a comprehensive suite for generating binary optimization problems with planted solutions. *arXiv preprint arXiv:2005.14344* (2020).

[24] Sherrington, D. & Kirkpatrick, S. Solvable model of a spin-glass. *Phys. Rev. Lett.* **35**, 1792–1796 (1975).

[25] Wang, W., Machta, J. & Katzgraber, H. G. Comparing monte carlo methods for finding ground states of ising spin glasses: Population annealing, simulated annealing, and parallel tempering. *Physical Review E* **92**, 013303 (2015).

[26] De Simone, C. *et al.* Exact ground states of ising spin glasses: New experimental results with a branch-and-cut algorithm. *Journal of Statistical Physics* **80**, 487–496 (1995).

[27] Barahona, F. On the computational complexity of ising spin glass models. *Journal of Physics A: Mathematical and General* **15**, 3241 (1982).

[28] Hartmann, A. K. Ground states of two-dimensional ising spin glasses: fast algorithms, recent developments and a ferromagnet-spin glass mixture. *Journal of Statistical Physics* **144**, 519

(2011).

[29] Khoshbakht, H. & Weigel, M. Domain-wall excitations in the two-dimensional ising spin glass. *Physical Review B* **97**, 064410 (2018).

[30] Kolmogorov, V. Blossom v: A new implementation of a minimum cost perfect matching algorithm. *Mathematical Programming Computation* **1**, 43–67 (2009).

[31] Robert, C. P. & Casella, G. The metropolis—hastings algorithm. In *Monte Carlo Statistical Methods*, 231–283 (Springer, 1999).

[32] Kirkpatrick, S., Gelatt, C. D. & Vecchi, M. P. Optimization by simulated annealing. *Science* **220**, 671–680 (1983).

[33] Gubernatis, J. E. The monte carlo method in the physical sciences: celebrating the 50th anniversary of the metropolis algorithm. *The Monte Carlo Method in the Physical Sciences* **690** (2003).

[34] Swendsen, R. H. & Wang, J.-S. Replica monte carlo simulation of spin-glasses. *Physical Review Letters* **57**, 2607 (1986).

[35] Geyer, C. J. *et al.* Computing science and statistics: Proceedings of the 23rd symposium on the interface. *American Statistical Association, New York* **156** (1991).

[36] Hukushima, K. & Nemoto, K. Exchange monte carlo method and application to spin glass simulations. *Journal of the Physical Society of Japan* **65**, 1604–1608 (1996).

[37] Mills, K., Ronagh, P. & Tamblyn, I. Finding the ground state of spin hamiltonians with reinforcement learning. *Nature Machine Intelligence* **2**, 509–517 (2020).

[38] Pahng, S. H. & Brenner, M. P. Predicting ground state configuration of energy landscape ensemble using graph neural network. In *Second Workshop on Machine Learning and the Physical Sciences (NeurIPS)* (2019).

[39] Zhou, H.-J. & Zheng, W.-M. Loop-corrected belief propagation for lattice spin models. *The European Physical Journal B* **88**, 1–10 (2015).
