# Supplementary material for: Searching for spin glass ground states through deep reinforcement learning
Source: Nat Commun. 2023 Feb 9;14:725. doi: 10.1038/s41467-023-36363-w (PMC9911406; doi:10.1038/s41467-023-36363-w)
Supplement: Supplementary file 3 — Description of additional Supplementary File [file 41467_2023_36363_MOESM3_ESM.pdf]

Descriptions of additional supplementary files

Supplementary Movie 1: In this movie, we show the whole decision process for DIRAC on an anti-ferromagnetic model. It started with the all-spin-up configuration. For each step, a spin with the maximal Q value was selected to flip. The red spins are the flipped ones; for other spins, the darker ones correspond to higher Q values. At step 200, DIRAC found the ground state. DIRAC did NOT make any wrong decision during the whole process.

Supplementary Movie 2: In this movie, we show the whole decision process for the greedy algorithm on an anti-ferromagnetic model. It started with the all-spin-up configuration. For each step, a spin with the maximal energy was selected to flip. The red spins are the flipped ones; for other spins, the darker ones correspond to higher spin energies. At step 191, the greedy algorithm stopped as there is no more spin with positive energy to be flipped.

Supplementary Movie 3: In this movie, we show the whole decision process for the SA algorithm on an anti-ferromagnetic model. It started with the all-spin-up configuration.For each step, a spin with the maximal energy was selected to flip. The red spins are the flipped ones; for other spins, the darker ones correspond to higher spin energies. At step 21,333, the SA finally found the ground state.

Supplementary Movie 4: In this movie, we show the whole decision process for the PT algorithm on an anti-ferromagnetic model. It started with the all-spin-up configuration. For each step, a spin with the maximal energy was selected to flip. The red spins are the flipped ones; for other spins, the darker ones correspond to higher spin energies. At step 10,808, the PT algorithm finally found the ground state.
